# Evidence for specificity of polygenic contributions to attainment in English, maths and science during adolescence

**DOI:** 10.1038/s41598-021-82877-y

**Published:** 2021-02-16

**Authors:** Georgina Donati, Iroise Dumontheil, Oliver Pain, Kathryn Asbury, Emma L. Meaburn

**Affiliations:** 1 Centre for Brain and Cognitive Development, Department of Psychological Sciences, Birkbeck, University of London, London, UK; 2 Centre for Educational Neuroscience, University of London, London, UK; 3 Social Genetic and Developmental Psychology, Institute of Psychiatry, Psychology and Neuroscience, King’s College London, London, UK; 4 Department of Education, University of York, York, UK

**Keywords:** Genome-wide association studies, Genetic association study, Behavioural genetics

## Abstract

How well one does at school is predictive of a wide range of important cognitive, socioeconomic, and health outcomes. The last few years have shown marked advancement in our understanding of the genetic contributions to, and correlations with, academic attainment. However, there exists a gap in our understanding of the specificity of genetic associations with performance in academic subjects during adolescence, a critical developmental period. To address this, the Avon Longitudinal Study of Parents and Children was used to conduct genome-wide association studies of standardised national English (N = 5983), maths (N = 6017) and science (N = 6089) tests. High SNP-based heritabilities (h^2^_SNP_) for all subjects were found (41–53%). Further, h^2^_SNP_ for maths and science remained after removing shared variance between subjects or IQ (N = 3197–5895). One genome-wide significant single nucleotide polymorphism (rs952964, p = 4.86 × 10^–8^) and four gene-level associations with science attainment (*MEF2C, BRINP1*, *S100A1* and *S100A13*) were identified. Rs952964 remained significant after removing the variance shared between academic subjects. The findings highlight the benefits of using environmentally homogeneous samples for genetic analyses and indicate that finer-grained phenotyping will help build more specific biological models of variance in learning processes and abilities.

## Introduction

Academic attainment (broadly defined as performance in educational benchmarks such as national exams and curriculum assessments; AA) consistently and reliably associates with a diverse array of emotional, cognitive and health outcomes^1,2^. As a consequence, identifying the contributions of specific genetic (and environmental) factors to individual differences in AA and understanding the aetiology of these observed relationships could be informative for a range of societal issues.

A large body of twin and DNA-based research robustly demonstrates that AA and learning abilities are heritable, with a recent meta-analysis of 61 twin studies reporting a heritability estimate of 66% for AA^3^. The first genome-wide association studies (GWAS) of learning abilities using modest (< 2500) sample sizes demonstrated that they are highly polygenic with thousands of common DNA variants contributing to heritability^4–6^. Furthermore, both twin and DNA-based studies suggest that the same genetic variants influence variability in cognitive functions across domains—dubbed ‘generalists genes’^7^. As a consequence, there has been a shift away from examining finer-grained measures of cognitive processes towards performing population-scale GWAS of broad measures of academic attainment, primarily how long one spends in education (EduYrs). The first EduYrs GWAS (N = 126,559) was a meta-analysis of 42 cohorts performed by the Social Science Genetic Association Consortium (SSGAC; thessgac.org). Three independent genome-wide significant single nucleotide polymorphisms (SNPs) were identified, each accounting for around 0.02% of the variance in EduYrs. Functional interrogation revealed that the signals were located in (or close to) genes previously associated with health and cognition, and demonstrated the utility of taking a broad phenotype to maximise discovery of common genetic variants of very small effect^8,9^. The subsequent SSGAC GWAS (EduYrs2; N = 293,723) identified a further 74 SNP associations that were mostly located in regions involved in the regulation of gene expression during foetal brain development^10^. The most recent SSGAC GWAS comprised over 1 million people (EduYrs3) and reported 1271 significantly associated loci and an enrichment of genes involved in neurophysiological brain functions. These included genes involved in neurotransmitter secretion, the activation of ion channels, synaptic plasticity, as well as those expressed in neural tissue both pre- and postnatally^11^. Construction of an EduYrs genome-wide polygenic score (GPS; a score representing an individual’s amalgamated genetic liability derived from the GWAS results) was predictive of 7–10% of the variance in general cognitive ability and 11–13% in educational attainment, making it one of the most predictive polygenic scores currently available in the behavioural sciences^11^. The EduYrs GPS associates with many phenotypes, which is perhaps not surprising: such a broad-brush measure taken in adulthood (many levels removed from cell structure and brain function) will necessarily capture a wide range of variables influencing the length of time people stay in formal education. For example, beyond cognitive ability the EduYrs GPS predicts traits such as child behavioural problems, negative symptoms of affect related to depression, callous and unemotional traits, and Attention Deficit Hyperactivity Disorder (ADHD)^12,13^. If these polygenic scores are to have practical applications, we need a much better understanding of the specificity and generality of genetic causes of variance in educational performance across development.

Whilst studies are limited, there is evidence of specificity in the genetic profiles of individual academic subjects and cognitive abilities. Donati, Meaburn and Dumontheil demonstrated that a structural equation model that divides AA into separate academic subjects fits standardised national test score data better than a common AA model, and that there is cognitive specificity to attainment in different subjects^14^. Consistent with these findings, bivariate twin estimates of English, maths and science attainment at ages 7–16 years suggest that just over half to two thirds of the heritability is shared between subjects after controlling for IQ [maths–English (0.54), science–English (0.64), science–maths (0.69)], leaving a considerable proportion of the heritability *not shared* across subjects and independent of general cognitive function^15^. Similarly, in a study of 13,262 12-year-old twins, maths retained a heritability of 0.44 when controlling for both general intelligence and reading ability^16^.

Taken together, these findings highlight the need to maintain specificity when investigating genetic predictors of academic attainment, and to consider the temporality of associations across childhood and adolescence. However, adequately powered molecular genetic studies focused on characterising genetic contributions to performance in individual academic subjects and their cognitive and non-cognitive genetic correlates are sparse. For instance, published GWAS of maths ability using general population samples have been limited in size (N = 602 to 3000 individuals) and thus far have failed to identify robustly associated variants^4,17^. An exception is a recent GWAS of Han Chinese samples (combined N = 1597) that reported four independent SNPs associated with maths ability^18^, although these have yet to be independently replicated. The largest maths GWAS to date used adult self-report retrospective measures of maths ability (N = 564,698) and highest maths class completed (N = 430,445), and found genetic correlations of 0.51 and 0.81 respectively with EduYrs3^11^. A multivariate twin and DNA-based analysis of reading and maths ability at age 12 in a UK twin cohort failed to identify any genome-wide significant loci but reported a SNP-based genetic correlation of 0.74 (although the exact magnitude of shared genetic influence was difficult to ascertain due to the large standard error)^16^. We are not aware of published GWAS that examine English or science as discrete academic subjects, but note that a large GWAS of latent cognitive domains captured by school grades in Danish, English and maths is in preprint^19^. GWAS of reading ability and receptive language (both components of English performance) have been reported^5,20^.

Untangling the generality, specificity and developmental timing of genetic causes of variance in discrete educational outcomes is necessary for understanding causal paths and will have far-reaching implications for the development of polygenic predictors and intervention strategies aimed at improving learning outcomes. To date, there have been no published GWAS for standardized attainment scores in English and maths during adolescence, and no GWAS at all for science. Adolescence is a crucial developmental period during which individuals’ executive function continues to mature and they are able to learn increasingly abstract and complex concepts^21^. Furthermore, a diverse range of cognitive and non-cognitive traits including well-being, personality and behavioural problems have been shown to contribute to the heritability of adolescent academic performance^22^.

This study aimed to assess whether there exist academic subject-specific molecular genetic contributions to English, maths and science—signal over and above that found for generalist genetic effects. This was approached in three ways: Firstly we performed three univariate GWA analyses of English, maths and science attainment to identify SNP-AA associations and estimate SNP-based heritability. Secondly to further explore the genetic contributions to subject-specific variance, we performed two further GWAS for each subject, one with the shared variance with the other subjects removed, and one with the shared variance with IQ removed. These GWAS were performed separately to assess whether subjects shared genetic variance over and above that shared with IQ. Thirdly, we used publicly available GWAS summary statistics from independent studies to estimate pairwise genetic correlations between academic attainment and 13 cognitive and non-cognitive traits and disorders in order to explore the magnitude and specificity of genetic correlations between individual academic subjects and related cognitive, mental health and personality traits. We predicted that (1) we would find both differences and commonalities in the outcomes of the three GWAS, (2) that there would exist some residual subject-specific SNP-based heritability after removing effects of generalist genes, and (3) that genetic correlations between the three academic subjects and the 13 related traits would vary in magnitude.

## Results

### Genome-wide association analyses of attainment in English, science and maths

**Figure 1:**
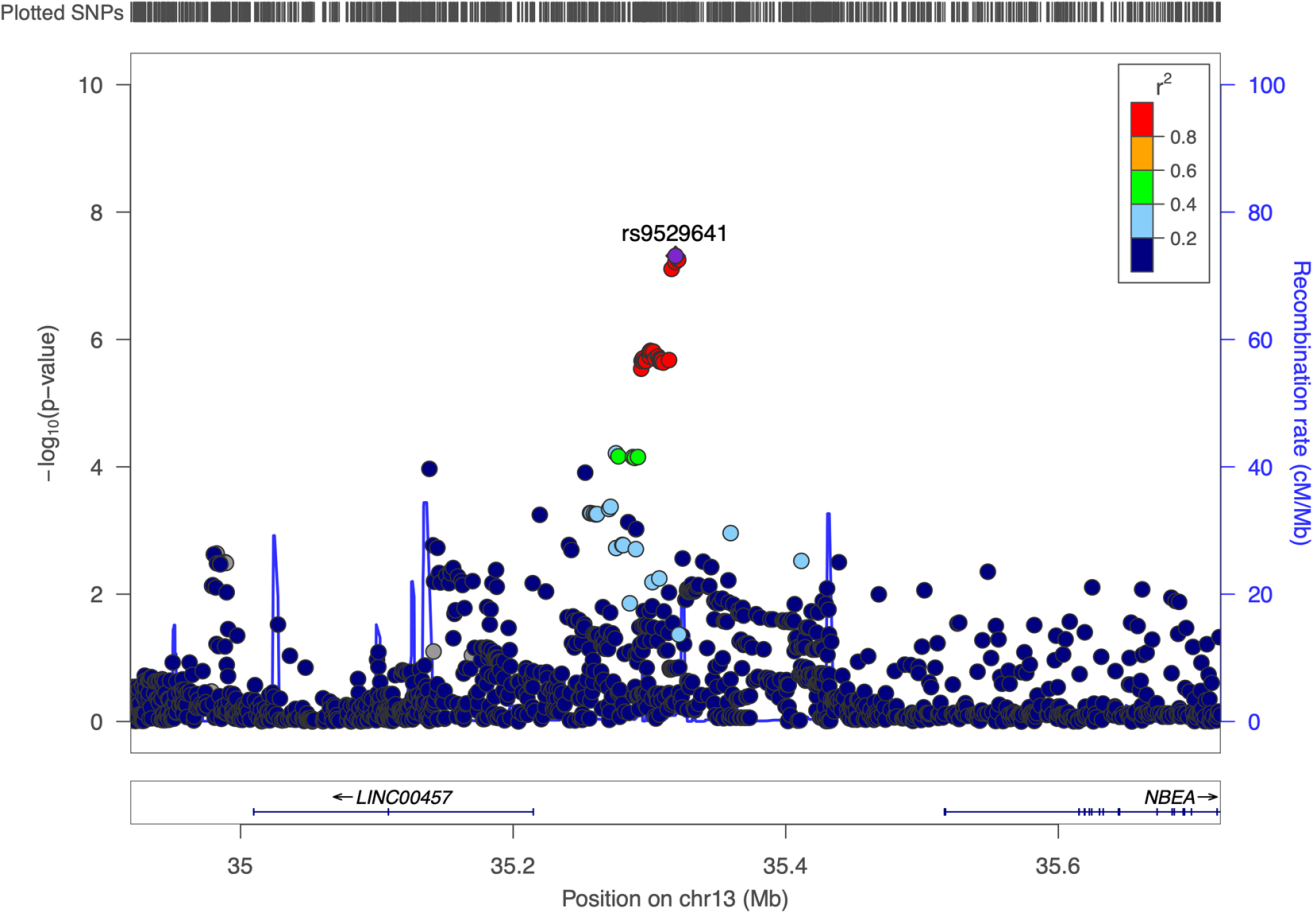
Locus Zoom plot of the SNP rs952964 (± 200 kb) on chromosome 13 [position: 35,319,175, minor allele frequency: 0.25, beta: 0.12 (0.02)] showing genome-wide significant association with science attainment during adolescence (11–14 years). The y-axis shows the p-value and the x-axis shows position on chromosome 13. Points show other SNPs located in this region—the purple SNP is the lead SNP and the other colours show the level of LD shared with the lead SNP.

Children in the UK-based ALSPAC cohort sat obligatory National Curriculum-based Standardised Assessment tests (SATs) at 11 and 14 years of age, designed to assess performance in the academic subjects of English, maths and science. In the current study academic attainment scores for English (N = 5983), maths (N = 6017) and science (N = 6089) were calculated by summing age- and sex-regressed SAT scores from these two time points for each academic subject (see “Methods”). Genome-wide SNP genotyping data is available for the ALSPAC sample and following quality control procedures 6,319,684 SNPs (MAF ≥ 1%) were used to perform three independent GWAS of English, maths and science (see “Methods” and Supplementary Notes 1 and 2). Across the three GWAS we identified one genome-wide significant SNP association for attainment in science (rs9529641, p = 4.86 × 10^–8^) (Fig. 1, see also Supplementary Fig. S1A and Supplementary Table S2), but none for attainment in English or maths (Supplementary Fig. S1B–C). A further 26 independent SNPs showed suggestive evidence of association (p ≤ 1 × 10^–5^) with science, 38 for maths and 16 for English (Supplementary Tables S3–S5). Quantile–quantile plots demonstrate an inflation of low p-values for science and maths, and inspection of linkage disequilibrium (LD) score regression intercept suggests this was not due to the presence of population stratification (intercept = 1.01–1.03) (Supplementary Fig. S1).

### Gene-based association analyses

In order to gain insight into functional pathways associated with AA we performed gene and gene-set analysis in MAGMA^23^ (“Methods”). Results of the genome-wide gene-based test for association are shown in Table 1 and Supplementary Figs. S3 and S4. In total four genome-wide significant (p = 0.05/17,875 genes tested = 2.80 × 10^–6^) gene-based associations with science were identified: *MEF2C* (Myocyte Enhancer Factor 2C; p = 2 × 10^–8^), *BRINP1* (Bone Morphogenetic Protein/Retinoic Acid Inducible Neural-Specific 1; p = 4 × 10^–7^), *S100A1* (S100 Calcium Binding Protein A1; p = 8 × 10^–7^) and *S100A13* (S100 Calcium Binding Protein; p = 1 × 10^–6^). No significant gene-based associations were found for maths or English. We further examined gene expression patterns for the four genes using the Genotype-Tissue Expression (GTEx) portal (https://gtexportal.org; Table 1, Supplementary Figs. S5–S8). Gene-set analysis revealed two gene-sets significantly (p ≤ 0.05/10,673) associated with English, and one with science (Supplementary Table S6). For full gene-based results see Supplementary Data 2.

**Table 1:** Significant gene-level associations with science attainment scores.

Chr	Position	Ensembl gene ID	Gene name	p-value	z-statistic	Gene expression^a^
1	153,600,873–153,604,513	ENSG00000160678	*S100A1*	**8.32 × 10**^**–7**^	4.790	Mostly brain
1	153,591,275–153,606,582	ENSG00000189171	*S100A13*	**1.16 × 10**^**–6**^	4.723	Broadly throughout the body
5	88,014,058–88,199,922	ENSG00000078725	*MEF2C*	**2.39 × 10**^**–8**^	5.459	Primarily in brain, lymphocytes and musculature
9	121,928,907– 122,131,739	ENSG00000081189	*BRINP1*	**3.82 × 10**^**–7**^	4.944	Almost entirely brain

Position based on GRCh37. Bolded figures show significant associations (p < 2.80 × 10^−6^)

^a^Expression data examined using the GTEx portal on 22/08/2018.

### Replication of SNP and gene-level associations

Replication of the SNP and gene associations was performed using data from the Twins Early Development Study (TEDS, N = 2330)^24^. We failed to replicate the genome-wide significant SNP (rs9529641) and gene-level associations for science attainment in this independent longitudinal cohort, although the smaller sample and different phenotype complicates interpretation (see “Methods” and Supplementary Note 3). We also cross-referenced our SNP and gene associations with two closely related GWAS^11,25^. Rs9529641 was significantly associated with both EduYrs3 and Intelligence (Table 2). At the gene-level *MEFC2* was also significantly associated with both EduYrs3 and Intelligence, and *BRINP1 * and *S100A13* with EduYrs3 (Table 2).

**Table 2:** Table of SNP and gene-level replication in TEDS, EduYrs3 and Intelligence GWAS.

Chr	SNP/Gene	P (ALSPAC) N = 6089	P (TEDS) N = 2330	P (EduYrs3)^a^ N = 1.1mil	P (Intelligence)^a^ N = 78,308
13	rs9529641	**4.86 × 10**^**–8**^	0.417	**1.53 × 10**^**–3**^	**3.98 × 10**^**–5**^
1	*S100A1*	**4.61 × 10**^**–7**^	0.484	NA	0.761
1	*S100A13*	**5.71 × 10**^**–7**^	0.513	**0.015**	0.786
9	*BRINP1*	**4.23 × 10**^**–7**^	0.240	**1.89 × 10**^**–10**^	0.557
5	*MEF2C*	**2.29 × 10**^**–8**^	0.907	**7.31 × 10**^**–39**^	**6.79 × 10**^**–13**^

^a^Results were extracted from the Supplementary Tables provided in the papers and included all participants. Bolded figures show significant associations (p < 0.05).

### SNP-based heritability and bivariate genetic correlations of AA

We employed two statistical methods to provide complementary estimates of SNP heritability (h^2^_SNP_) for science, maths, and English: LD-score regression (LDSC^26^) and Genome-Based Restricted Maximum Likelihood (GREML) analysis^27^. LDSC regression h^2^_SNP_ estimates using GWAS summary statistics were moderate and significant for all three academic subjects (LDSC unconstrained h^2^_SNP_ = 0.29–0.34, constrained h^2^_SNP_ = 0.36–0.54), with the highest estimate obtained for science (Table 3, Supplementary Table S7). GREML h^2^_SNP_ estimates were similar in magnitude, ranging from 0.41 (English) to 0.53 (science) (Table S7).

**Table 3:** SNP-based heritability estimates, and genetic and phenotypic correlations between academic subjects.

LDSC h^2^_SNP_	English	Maths	Science
English	0.360 (0.058)***	0.691 (0.005)***	0.732 (0.005)***
Maths	0.745 (0.141)***^a^	0.473 (0.058)***	0.811 (0.003)***
Science	0.722 (0.141)***^a^	0.620 (0.121)***^a^	0.535 (0.058)***

Genetic correlations are presented below the diagonal (bold), SNP-based heritability on the diagonal (underline) and phenotypic correlations above the diagonal (italics). Standard errors are in parentheses. Due to the genetically homogenous nature of this sample LDSC SNP heritability and genetic correlation estimates are reported with the h^2^ intercept constrained to 1. Unconstrained LDSC estimates are provided in Supplementary Table S7. Phenotypically, science is significantly more correlated with English (z = 5.80, p = 3.35 × 10^–9^) and maths (z = 20.58, p = 2.08 × 10^–94^) than these were with each other. There are no significant differences in the genetic correlations between subjects (p’s > 0.05).

***p ≤ 0.001.

^a^These correlations were significantly less than 1 (Maths–English, p = 0.04; Science–English, p = 0.03; Science–Maths, p = 0.001).

Bivariate genetic correlations (r_g_) between academic subjects estimated by LDSC regression performed in Unix were high (r_g_ = 0.62–0.75) but significantly less than 1, illustrating a degree of genetic specificity (Table 3).

### AA and IQ regressed analyses

Academic attainment across subjects is correlated at both the phenotypic and genetic level (Tables 3 and S1), and previous research demonstrates school performance is highly correlated with general cognitive function, an association partly underpinned by generalist genes^7^. We therefore sought to examine genetic contributions to *subject-specific* variance independent of performance in the other two subjects, or independent of IQ. This was achieved by regressing verbal and non-verbal IQ from the academic attainment scores for English (N = 3,197, verbal r = 0.548 SE = 0.011; non-verbal r = 0.209, SE = 0.015), maths (N = 3212, verbal r = 0.491, SE = 0.012; non-verbal r = 0.298, SE = 0.015) and science (N = 3260, verbal r = 0.587, SE = 0.010; non-verbal r = 0.269, SE = 0.015), or regressing out attainment in the other two subjects (N = 5895) (see “Methods”). Univariate GWAS of the AA-regressed and IQ-regressed attainment measures for each subject failed to identify any genome wide significant associations (Supplementary Fig. S9). LDSC-based h^2^_SNP_ estimates were lower than in the original analyses, but still significantly larger than 0 for AA-regressed and IQ-regressed science and maths scores (p′s < 0.05) (Table 4). GREML h^2^_SNP_ estimates were similar, ranging from 0.08 (AAreg-English) to 0.21 (IQreg-maths) (Supplementary Table S7).

**Table 4:** SNP-based heritability (h^2^_SNP_) estimates for academic subjects after controlling for attainment in other subjects (AAreg), and after controlling for IQ (IQreg).

	English (AAreg)	Maths (AAreg)	Science (AAreg)	English (IQreg)	Maths (IQreg)	Science (IQreg)
h^2^_SNP_	0.06 (0.06)	0.12 (0.06)*	0.13 (0.05)**	0.07 (0.11)	0.24 (0.11)*	0.15 (0.09)*

SNP-based heritabilities were estimated using LD score regression with h^2^ intercepts constrained to 1. Bolded estimates show those that are significantly greater than 0, *p < 0.05, ** p < 0.01. Standard errors are in parentheses.

Finally, in order to check the specificity of the SNP association identified with science, we performed a hypothesis-driven lookup in the AAreg and IQreg GWAS results (Table 5) using a Bonferroni corrected p-value threshold of 0.05/6 per SNP/gene. The SNP rs9529641 remained associated with science attainment only in the AA regressed GWAS (p = 0.001). None of the gene-level associations with science were significantly associated with AAreg or IQreg science after Bonferroni correction (AAreg science *S100A1*: z = 1.9, p = 0.03; *S100A13*: z = 1.3, p = 0.10; *MEF2C*: z = 2.3, p = 0.01; *BRINP1*: z = 2.2, p = 0.01; IQreg science *S100A1*: z = 1.3, p = 0.10; *S100A13*, z = 0.8, p = 0.19; *MEF2C*: z = 1.7, p = 0.05, *BRINP1*: z = 1.8, p = 0.03).

**Table 5:** Associations between rs9529641 and all nine GWAS phenotypes.

GWAS	Beta	SE	P-value
**Science**	**0.12**	**0.021**	**4.86 × 10**^**–08**^
Maths	0.09	0.021	4.59 × 10^–05^
English	0.05	0.021	0.011
**AA reg Science**	**0.07**	**0.021**	**0.001**
AA reg Maths	0.01	0.021	0.539
AA reg English	-0.03	0.021	0.252
IQ reg Science	0.08	0.029	0.010
IQ reg Maths	0.04	0.029	0.146
IQ reg English	0.01	0.029	0.787

Bolded figures show significant associations. For the first set of analyses genome-wide significance is p ≤ 5 × 10^–8^. Subsequent tests were hypothesis driven and therefore Bonferroni corrected to p ≤ 0.008 (0.05/6).

Using a z-test we were able to show that rs9529641 is significantly more associated with science than English (z = 2.48, p = 0.01) but not maths (z = 1.06, p = 0.14). It was significantly more associated with AAreg science than both AAreg English (z = 3.54, p < 0.001) and AAreg maths (z = 2.12, p = 0.02), and it was also significantly more associated with IQreg science than IQreg English (z = 1.65, p = 0.05), but not IQreg maths (z = 0.94, p = 0.17).

### Genetic correlations between AA and related phenotypes

We estimated genetic correlations between subject attainment scores and the GWAS summary statistics of 13 cognitive, educational, psychiatric and personality phenotypes available in the LD hub resource for European samples (http://ldsc.broadinstitute.org/ldhub/). Table 6 shows the magnitude and direction of the genetic relationships for all three academic traits. As expected, genetic correlations with adult academic attainment (years of schooling) and general intelligence were consistently high and positive (r_g_ = 0.89 to 1.26). Genetic correlations with personality and psychiatric traits were lower, with some variation across subjects, although not statistically different after correction (Supplementary Table S8 for full details).

**Table 6:**
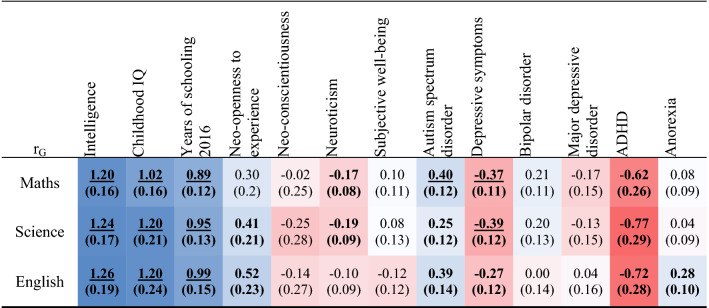
LDSC-based genetic correlations between English, maths and science and 13 related educational and psychological traits.

See Supplementary Table S8 for more details.

Bolded correlation coefficients are significant p < 0.05 (uncorrected). Underlined are significant after Bonferroni correction p $\le$ 0.001 (0.05/39). Red cells represent negative correlations and blue cells positive correlations. Intercepts were not constrained in any of these analyses. Statistical differences in correlation coefficients between academic subjects and a given trait were tested with a z-test; none of the correlations were significantly different from one another. Estimates > 1 are not uncommon in LDSC regression particularly if there is some sample overlap and suggests that the estimation is close to 1 with error.

## Discussion

This study reports the first GWAS of science attainment and the largest published GWAS of maths and English attainment using national standardised tests. The current dominant framework for assessing the genetic contributions to variance in academic attainment and its relationship to other outcomes uses a broad measure of attainment^11^, which prevents interpretation of the specificity of the relationships identified. We sought to overcome this issue by differentiating between performance in the academic subjects of English, maths and science using national standardised tests of performance to investigate the degree of genetic specificity and overlap with other cognitive, educational, psychiatric and personality traits. Despite clear evidence for the role of general mechanisms in building the brain, the brain also supports different functional operations (e.g. counting vs reading), which must be supported by a degree of genetic specificity. To understand these mechanisms better, analyses of more specific phenotypes are needed.

The three GWAS of maths, science and English found one genome-wide significant SNP (rs9529641) associated with science attainment. Rs9529641 is not an expression quantitative trait loci, i.e. it does not influence gene expression, but the nearby gene *NBEA* is largely expressed in the brain, and de novo variants in *NBEA* have been reported in neurodevelopmental cohorts^28^. Rs9529641 was also nominally associated with maths (p = 0.0001), but not English (p = 0.011) leaving open the possibility that the difference in p-values between the subjects is due to chance and the slightly different sample sizes, however the significant differences in effect sizes suggests otherwise. Rs9529641 also reaches p ≤ 0.001 in both the EduYrs3^11^ and Intelligence^25^ large GWAS meta-analyses. Furthermore, after regressing out variance explained by performance in the other two academic subjects, this association remained significant and specific to science. What predicts variance in science academic achievement is an understudied topic compared to maths and English. Research to-date suggests a pattern similar to the genetic results found here, with considerable similarities but also some subtle differences in the patterns of association with executive function skills, vocabulary and IQ^14,29^. There is some evidence that science achievement may be more dependent than mathematics or English on central executive working memory processes and more complex aspects of EF, such as planning^29,30^. When regressing out IQ, the science association with rs9529641 was not significant after correction for multiple testing, however the effect size remained the same. Although the SNP association with science failed to replicate in the independent Twins Early Development Study sample, we note the relatively small sample available and that the TEDS attainment measures were based on teacher ratings and not standardised across participants.

Gene-based association analyses identified four genes associated with attainment in science, but none for maths or English. The strongest signal came from the *MEF2C* gene, which also shows strong evidence of association in the EduYrs3 and Intelligence GWAS studies. Notably, studies looking at general cognitive ability have sought *MEF2C* associations in hypothesis driven tests^31^ and more recently it has been associated with a large meta-analysis of intelligence and years in education^32^ and the largest depression GWAS to date^33^. *MEF2C*, located on chromosome 5, has been linked to synaptic plasticity, memory and learning^34^. It is primarily expressed in the brain and haploinsufficiency of *MEF2C* is associated with severe cognitive impairment, stereotypic movements, epilepsy and cerebral malformation^35^. Evidence from animal models also suggests that it is involved in the development of memory and the consolidation of information^34^. Over-expression of *MEF2C* has also been implicated in poor developmental and cognitive outcomes^36,37^ and it has been associated with Alzheimer’s disease^38^. Given its apparently more general role in cognition, and the lower but not trivial associations with maths (p = 5.12 × 10^–6^) and English (p = 9.76 × 10^–5^), we are not suggesting *MEF2C* is a science specific gene. Rather, we suggest there may be evidence for a ‘dosage effect’, where the brain mechanisms that are built through the *MEFC2* gene may explain more variance in science than in English and maths. A similar effect may be at play with the rs9529641, where the mechanism linked to this SNP leads to greater individual differences in science than in English and maths.

The second most strongly associated gene was *BRINP1*, which is also primarily expressed in the brain, and is involved in protein binding. *BRINP1* has been involved in a wide range of processes related to cognition and behaviour^39,40^. The final two associated genes were *S100A1* and *S100A13*, both of which are members of the S100 protein family that encode calcium binding proteins and are involved in the regulation of a wide range of intra- and extracellular processes. These include cell cycle progression, differentiation and possibly stimulation of Ca^2+^ release^41,42^. After correction for multiple testing, none of the four genes were significantly associated with science after regressing out variance explained by maths and English or IQ.

SNP-based heritability estimates were moderate for all three subjects, and substantially closer to the twin-based heritability estimates of 65% for maths (h^2^_SNP_ = 47%) and 54% for science (h^2^_SNP_ = 54%)^15^ than is often the case with DNA-based estimates (e.g. maths ability h^2^_SNP_ = 0.16^11^). This is unusual because twin estimates capture all additive genetic effects that contribute to a phenotype, whereas GWAS estimates include only additive effects of common SNPs. These high estimates might be driven by the homogenous nature of the sample, both environmentally and ancestrally, as well as the use of a standardised assessment of academic ability. Moreover, they suggest that the majority of the genetic variance contributing to individual differences in academic attainment in adolescence comes from the additive effects of common, rather than rare, genetic variation.

We found a significant overlap of common genetic variants influencing variability in the three academic subjects, indicated by the large, but significantly smaller than 1, genetic correlations (r_g_ = 0.62–0.75). The degree of specificity estimated by looking at genetic correlations using LDscore indicate a moderate to small but potentially informative degree of specificity. In order to confirm this, we performed further GWA analyses of each subject with the variance of the other two subjects removed and controlling for IQ. Maths and science maintained heritability significantly higher than 0 in both instances. Interestingly the IQ regressed measures retained more heritability than the AA regressed measures suggesting that the subject attainment measures shared more heritable variance with each other than with IQ.

Phenotypically, science was significantly more correlated with English and maths than these were with each other (Table 3), suggesting academic performance in science might incorporate variance from the other two subjects^43^. Whilst no significant differences in genetic correlations between academic subjects were found, we note the opposite pattern of associations, with English and maths being the most highly genetically correlated. One possible explanation is that the factors contributing to the phenotypic correlation in performance between science and maths, and science and English, are under greater environmental influence than the factors contributing to the correlation between English and maths performance, which correlate more for genetic reasons. Note, it is possible that this is a specific effect of this period of development and may not be found earlier or later.

Genetic correlations with cognitive and academic attainment in other studies were found to be high and consistent across the academic subjects. Genetic correlations with personality traits varied more across academic subjects, although these differences in estimates were not statistically significant. Associations between cognition and mental health have been noted in a number of genetic and non-genetic studies^44,45^. The fact that pairwise genetic correlations between AA and autism, and AA and ADHD go in opposite directions is interesting because recent work has shown a positive shared genetic basis to ASD and ADHD symptomology in the ALSPAC sample^46^. Depression and anorexia represent another pair of traits that have been found to be correlated phenotypically and genetically in twin studies^47^, but each show opposite directions of genetic overlap with the three academic subjects. Further analysis using multivariate models would be needed to directly assess whether there are specific cognitive or academic abilities that may differentiate between these disorders.

Broadly, the results suggest that although there are underlying cognitive features which contribute to variance across all three academic subjects, there are other (both genetic and non-genetic) factors which contribute to subject-specific variance. These results reflect the conclusions of multivariate twin studies that examine genetic covariance between cognitive ability and subject attainment in a large UK twin cohort. For example, Kovas et al. (2005) investigated the genetic overlap between mathematics performance, reading and general intelligence in childhood. They reported considerable genetic correlations between mathematics and reading (r_gTWIN_ = 0.74) and between mathematics and ‘g’ (r_gTWIN_ = 0.67), but noted that approximately a third of the genetic variance in mathematics was independent of both of these factors, suggesting some degree of genetic specificity^7^. A subsequent study controlling for performance in maths, English and ‘g’ investigated the extent to which there was genetic specificity in science attainment in childhood^48^. The authors report a h^2^_twin_ of 49% for science and genetic effects beyond the other factors, which were therefore specific to science.

### Limitations

Whilst this study represents the largest GWAS to-date for science and English attainment, it is still underpowered to detect common variants of very small effect. Recently developed multivariate GWAS approaches such as genomic-structural equation modelling^49^ may help shed light on the specific causes of the observed genetic correlations identified. Furthermore, although this study used an adolescent sample, longitudinal genetic studies will be necessary to fully understand how genetic influences unfold over development. While we believe the use of a single, homogeneous, UK cohort allowed greater sensitivity to explore our research questions, it also limits the generalisability of the results to different populations. In particular, differences in schooling, such as a greater focus on drilling in mathematics, or differences intrinsic to the language spoken, such as grammatical complexity or spelling irregularities, may impact genetic associations with specific school subjects. However, if GWAS results are population specific due to variants becoming more relevant in particular contexts, perhaps in order for polygenic scores to be accurate, they will have to be population/environment specific. Finally, regressing out attainment in other subjects or IQ (both of which are heritable) risks inducing collider bias and distorting towards or away from true associations. However, we note that the focus of the AAreg and IQreg analyses were on exploring the specificity of science attainment associations, rather than identification of novel associations^50^.

## Conclusion

In this study, we performed a series of univariate GWAS of English, maths and science standardised national attainment scores, estimated SNP-based heritability and assessed shared genetic architecture with educational, cognitive, behavioural and psychiatric phenotypes. We found that rs9529641, *MEFC2* and *BRINP1* were significantly and robustly associated with science attainment. We also found differences in SNP-based heritability estimates and genetic correlations with other cognitive traits indicating, as with the phenotypic data, a degree of overlap and specificity between academic subjects. These findings suggest that understanding the sources of individual differences in academic attainment may facilitate a better understanding of the causal paths to later educational outcomes and mental health disorders. Future studies should examine these genetic relationships within a multivariate framework to allow the separation of general versus specific effects at the level of individual DNA sequence variants.

## Methods

### Participants

The Avon Longitudinal Study of Parents and Children (ALSPAC) (http://www.bristol.ac.uk/alspac/) is an on-going population-based study investigating factors influencing development and health. The study website contains details of all the data that is available through a fully searchable data dictionary (http://www.bristol.ac.uk/alspac/researchers/our-data/). Initial recruitment included 14,541 mothers with 13,988 children alive at age one. A second round of recruitment at around age 7 yielded a total sample size for data collected after this age of 15,247. See Supplementary Note 1 for more details and Boyd et al., and Fraser el al.,^51,52^. The sample for this study is comprised of children for whom data were available at both age 11 and 14 for English, maths or science attainment, along with genome-wide SNP genotyping data. Ethical approval for the study was obtained from the ALSPAC Ethics and Law Committee, and Birkbeck’s Department of Psychological Sciences Research Ethics committee. All research was performed in accordance with ALSPAC’s and Birkbeck’s Departmental relevant guidelines/regulations. Parents and/or legal guardians gave informed consent, and the authors had no access to any participant identifying information.

### Measures

Attainment in English, maths, and science was assessed using National Curriculum standardised tests at 11 and 14 years of age. At age 11 (end of Key Stage 2) and age 14 (end of Key Stage 3), national exams—known as the SATs—were obligatory in schools across the UK when these data were collected. Pupils sat the tests under exam conditions and scripts were externally marked, standardised, and given a curriculum level 1–9 (low to high).

At ages 11 and 14 the English SATs assess reading, grammar, punctuation and spelling, in addition to comprehension and interpretation of a studied text. Maths is assessed at both ages by written SATs that cover all areas of mathematics including conceptual understanding, mathematical reasoning and problem solving. At age 11, the maths SAT also includes a ‘mental maths’ component in which the students are asked questions orally and, under timed conditions, must record their answers having performed the computations in their head. The science SAT at ages 11 and 14 assesses the development of scientific thinking and knowledge, experimental skills and strategies, analysis and evaluation, scientific vocabulary, units, symbols and nomenclature.

To get the most reliable score of attainment only individuals with data at both 11 and 14 years (r = 0.67 to 0.81) were included. To remove variance associated with sex and age at testing, each academic subject score was first regressed on age and sex (at each time point) and the residuals from the linear regression were summed together to create a final score for each subject. This resulted in sample sizes of 5983 for English, 6017 for maths and 6089 for science.

To assess subject-specific genetics effects, AA regressed scores were created by removing the variance shared with the other two subjects from each of the final scores. This resulted in a sample of 5,895 individuals for each subject. Finally, in order to assess genetic effects independent of general cognitive ability, IQ regressed scores were created by removing the variance shared with IQ from each of the individual subject scores, leaving a smaller sample due to the lower availability of IQ scores than academic attainment measures (see Table 7). IQ was calculated using a combined measure of Vocabulary and Matrix Reasoning raw scores taken from the Wechsler Abbreviated Scale of Intelligence^53^ at age 15. In the Vocabulary subtest participants were asked the meaning of a list of gradually more complex words. The Matrix reasoning subtest consisted of a multiple-choice visual puzzle in which the participants were presented with a series of pictures and had to choose the missing image.

**Table 7:** Sample descriptive statistics.

Measure	N	N males	Range (SD)
English	5983	2909	− 2.50–1.48 (0.68)
Maths	6017	2950	− 2.77–1.90 (0.89)
Science	6089	2995	− 2.63–1.18 (0.73)
English-AA (with Maths + Science regressed out)	5895	2871	− 2.03–2.02 (0.48)
Maths-AA (with English + Science regressed out)	5895	2871	− 2.40–2.17 (0.59)
Science-AA (with English + Maths regressed out)	5895	2871	− 1.52–1.50 (0.44)
English-IQ (with IQ regressed out)	3197	1484	− 2.33–1.94 (0.56)
Maths-IQ (with IQ regressed out)	3212	1505	− 2.52–2.16 (0.73)
Science-IQ (with IQ regressed out)	3260	1531	− 2.64–1.75 (0.56)

Academic attainment scores are the sum of residuals from age and sex regressed linear models of individual SAT scores at age 11 (Key Stage 2) and 14 (Key Stage 3). Attainment in other subjects (e.g. English-AA) or IQ (e.g. English-IQ) were then regressed out to provide AA and IQ regressed scores to assess independent genetics effects.

### Genotyping and quality control

Genotyping and imputation were performed by ALSPAC. Adolescents from ALSPAC were genotyped using the Illumina HumanHap550 quad chip by 23andMe subcontracting the Wellcome Trust (Welcome Sanger Institute, Cambridge, UK) and the Laboratory Corporation of America (Burlington, NC, US). The raw genotype data were subjected to standard quality control procedures to identify individuals and SNPs for exclusion. Samples that passed quality control stages were phased and imputed using the Haplotype Reference Consortium panel of ~ 31,000 phased whole genomes and Impute V3^54^. SNP and sample quality control were repeated post-imputation (see Supplementary Note 2 for full details).

### Statistical analysis

All data preparation was performed using R 3.4^55^. Scores were regressed on the first 10 ancestry principal components to control for population structure and then quantile normalized in SNPtest^56^. In total nine GWA analyses were performed using (1) individual subject scores for attainment in English, maths and science, (2) AA regressed scores for attainment in English, maths and science (3), and IQ regressed scores for in English, maths and science. Each univariate GWA analysis was performed in SNPTest v.2 using an additive linear model and imputation probability calls^57^. Independent SNP association signals were identified by LD clumping in PLINKv1.9, with a genome-wide significance threshold for index SNPs and 0.2 threshold for LD clumping^58^.

Gene-based association analyses, which test for association between aggregated SNP effects across each gene, were performed using MAGMA within the FUMA programme, using the summary statistics from each GWAS^23,59^. Significantly associated genes were identified as those surviving Bonferroni correction for multiple testing (p = 0.05/17,875 genes tested = 2.80 × 10^–6^). Competitive gene-set analyses were also carried out in MAGMA using 10,673 gene sets (5915 GO terms, 4758 Curated gene sets) obtained from MsigDB v5.2. Functional interrogation of gene-based associations was conducted using the GTEx portal (https://gtexportal.org/home/).

The proportion of variance in science, maths or English accounted for by all the SNPs on the array passing QC, i.e. SNP heritability (h^2^_SNP_), was estimated using two methods that have differing modelling assumptions of the underlying genetic architecture^60^, with the view to gain consensus estimates of SNP heritability for academic attainment. GREML was implemented in the GCTA software package in Unix to provide h^2^_SNP_ estimates using individual level genetic data^27^. LD-score regression (LDSC^26^) in Unix was used to estimate h^2^_SNP_ using the GWAS summary statistics. Genetic correlations were estimated between cognitive, educational, psychiatric and personality traits available in—and using—LD hub (http://ldsc.broadinstitute.org). See Supplementary Note 4. Due to the homogeneous nature of the ALSPAC sample the LDSC h^2^ intercept was constrained to 1. We note that this will result in lower standard errors^26^ and also report unconstrained estimates. The AA-regressed and IQ-regressed attainment scores were excluded from this LDSC analysis due to the low SNP heritability estimates (and large standard errors) obtained leading to low heritability z-score (z < 4). Finally, z-tests were used to assess whether heritability results were significantly larger than 0, whether correlations were significantly smaller than 1 and whether correlations were significantly different from each other (p < 0.05).

### Replication

Replication of independent significantly associated SNPs and genes was performed using data from the Twins Early Development Study. TEDS is a longitudinal study investigating the cognitive and behavioural development of twins born in England and Wales between January 1994 and December 1996 (www.teds.ac.uk)^24^. TEDS participants completed various web and telephone-based tests and questionnaires at regular intervals over childhood and adolescence designed to assess various aspects of cognition, language and behaviour, which are described in detail elsewhere^61^. The available sample consisted of 2352 individuals (one member of each twin pair) for whom academic attainment data at age 14 and genome-wide SNP genotyping data were available (full details can be found in Supplementary Note 3). The TEDS cohort is a few years younger than the ALSPAC cohort (recruited 1994–1996), and as school exam procedures had changed during this time national exams (SATs) were no longer obligatory. Although Key Stage 3 (KS3; age 14) SAT assessments were given to some TEDS pupils, they were teacher rated, *not* nationally standardised. TEDS KS3 scores are therefore not directly comparable with ALSPAC scores and capture school and teacher effects. Phenotype and genotype data were retained for 2352 unrelated individuals for maths and 2330 for science. Linear genotype–phenotype regressions for SNP rs9529641 and the SNPs in genes S100A1, S100A13, BRINP1 and MEF2c were performed separately for each TEDS genotyping array platform (OEE or Affy), regressed on the first 10 ancestry principal components and were quantile normalized in SNPtest^57^. Platform-specific results were then meta-analysed using METAL^62^. Gene-level replication was performed using MAGMA^59^.

## Supplementary Information

Supplementary Information 1.

Supplementary Information 2.

## Data Availability

Requests for the genotypic and phenotypic datasets analysed and/or generated in the current study can be made via the ALSPAC website http://www.bristol.ac.uk/alspac/researchers/access/.
