# Supplementary material for: Evidence for specificity of polygenic contributions to attainment in English, maths and science during adolescence
Source: Sci Rep. 2021 Feb 16;11:3851. doi: 10.1038/s41598-021-82877-y (PMC7887196; doi:10.1038/s41598-021-82877-y)
Supplement: Supplementary file 1 — Supplementary Information 1. [file 41598_2021_82877_MOESM1_ESM.pdf]

Evidence for specificity of polygenic contributions to attainment in English, maths and science during adolescence

**Supplementary Information**

Georgina Donati[1,2], Iroise Dumontheil[1,2], Oliver Pain[3], Kathryn Asbury[4], Emma L. Meaburn*[1,2]

[1] Centre for Brain and Cognitive Development, Department of Psychological Sciences, Birkbeck, University of London, London, United Kingdom

[2] Centre for Educational Neuroscience, University of London, London, United Kingdom

[3] Social Genetic and Developmental Psychology, Institute of Psychiatry, Psychology and Neuroscience, King's College London, London, UK

[4] Department of Education, University of York, York, UK

*corresponding author: e.meaburn@bbk.ac.uk

# Supplementary Methods

**Supplementary Note 1**: ALSPAC cohort details

Pregnant women resident in Avon, UK, with expected dates of delivery 1st April 1991 to 31st December 1992 were invited to take part in the study. The initial number of pregnancies enrolled is 14,541 (for these at least one questionnaire has been returned or a "Children in Focus" clinic had been attended by 19/07/99). Of these initial pregnancies, there was a total of 14,676 foetuses, resulting in 14,062 live births and 13,988 children who were alive at 1 year of age.

When the oldest children were approximately 7 years of age, an attempt was made to bolster the initial sample with eligible cases who had failed to join the study originally. As a result, when considering variables collected from the age of seven onwards (and potentially abstracted from obstetric notes) there are data available for more than the 14,541 pregnancies mentioned above.

The number of new pregnancies not in the initial sample (known as Phase I enrolment) that are currently represented on the built files and reflecting enrolment status at the age of 18 is 706 (452 and 254 recruited during Phases II and III respectively), resulting in an additional 713 children being enrolled. The phases of enrolment are described in more detail in the cohort profile paper[1].

The total sample size for analyses using any data collected after the age of seven is therefore 15,247 pregnancies, resulting in 15,458 foetuses. Of this total sample of 15,458 foetuses, 14,775 were live births and 14,701 were alive at 1 year of age.

**Supplementary Note 2**: ALSPAC genotyping quality control details

ALSPAC children were genotyped using the Illumina HumanHap550 quad chip genotyping platforms by 23andme subcontracting the Wellcome Trust Sanger Institute, Cambridge, UK and the Laboratory Corporation of America, Burlington, NC, US. The resulting raw genome-wide data were subjected to standard quality control methods.

Individuals were removed on the basis of sex mismatches, excessive or low genetic heterozygosity (+/- 3SD), if individual missingness was > 3%, insufficient sample replication (IBD < 0.8), and if they were of non-European ancestry as measured using multidimensional scaling analyses compared with Hapmap II (release 22). SNPs were removed if they had a minor allele frequency < 1%, a call rate < 95%, or deviated from Hardy-Weinberg equilibrium (HWE, $p < 5 \times 10^{-7}$). Cryptic relatedness was measured as proportion of identity by descent (IBD > 0.1). Siblings that passed all other quality control thresholds were retained during subsequent phasing and imputation (and then removed in the final stages depending on phenotype availability or at random) to leave a final genetic sample of 9,115 participants and 500,527 SNPs. ALSPAC Mothers were genotyped (although not used in this study) and SNP data from mother-child duos were combined (477,482 SNPs in common) and a further 11,742 SNPs with genotype missingness above 1%, or that were out of HWE, were removed as well as a further 174 child participants, to leave 8,941 individuals. Haplotypes were estimated using ShapeIT (v2.r644) which utilises relatedness during phasing. The phased haplotypes were then imputed to the Haplotype Reference Consortium (HRC) panel of approximately 31,000 phased whole genomes. The HRC panel was phased using ShapeIt v2, and the imputation was performed using Impute V3. Imputed SNPs were excluded from all further analyses if they had a minor allele frequency < 0.01, an info score < 0.7, call rate < 0.95 and HWE $p < 5 \times 10^{-7}$, which resulted in a total of 6,319,684 SNPs. Ten principal components were created for the 8,872 *unrelated* individuals in order to control for population structure: using only observed (i.e., non-imputed) SNPs, short and long-range LD pruning was performed[2] and principal components were created using the 'pca' function in PLINKv1.9[3]. Principal components were plotted and four participants were identified as outliers and removed to leave a final genetic sample of 8,868 individuals.

**Supplementary Note 3**: Twins Early Development Study (TEDS) replication sample

School exam procedures changed between the recruitment of the ALSPAC and TEDS cohorts meaning that national exams (SATs) for the TEDS participants were no longer obligatory. Therefore, although Key Stage 3 (KS3; age 14) SAT assessments were given to some TEDS pupils, they were teacher rated rather than nationally standardised like the ALSPAC assessments and therefore likely capture school and teacher effects. Correlations between subject performance in the TEDS cohort are shown in **Table S1**.

KS3 scores for maths (range= -3.16 – 0.42) and science (range= -3.16 – 0.78) were regressed on age at time of examination and sex. Subject attainment scores and genotype data were retained for 2,352 unrelated individuals for maths (array platform: OEE = 1,363, Affy = 989) and 2,330 for science (OEE = 1,347, Affy = 983) as previously reported [14]. Linear genotype-phenotype regressions for SNP rs9529641 and the SNPS in genes *S100A1*, *S100A13*, *BRINP1* and *MEF2c* were performed separately for each array platform (OEE or Affy), regressed on the first 10 ancestry principal components and were quantile normalized in SNPtest[15]. Platform-specific results were then meta-analysed using METAL[16]. Gene-level replication was performed using MAGMA[17].

**Table S1:** TEDS academic subject performance phenotype correlations for KS3

| | Science | English | Maths |
|---|---|---|---|
| Science | 1 | | |
| English | 0.40 | 1 | |
| Maths | 0.48 | 0.38 | 1 |

Details of the TEDS genotype quality control are as follows. Genotyping of the TEDS cohort was performed in two waves. DNA from 3,747 samples was extracted from buccal cheek swabs and genotyped at Affymetrix, Santa Clara, California, USA. From this sample, 3,665 samples were successfully hybridized to AffymetrixGeneChip 6.0 SNP genotyping arrays (http://www.affymetrix.com/support/technical/datasheets/genomewide_snp6_datasheet.pdf) using experimental protocols recommended by the manufacturer (Affymetrix Inc., Santa Clara, CA). The raw image data from the arrays were normalized and pre-processed at the Wellcome Trust Sanger Institute, Hinxton, UK for genotyping as part of the Wellcome Trust Case Control Consortium 2 (https://www.wtccc.org.uk/ccc2/) according to the manufacturer's guidelines (http://www.affymetrix.com/support/downloads/manuals/genomewidesnp6_manual.pdf). Genotypes for the Affymetrix arrays were called using CHIAMO (https://mathgen.stats.ox.ac.uk/genetics_software/chiamo/chiamo.html).

DNA for 8,122 individuals (including 3,607 dizygotic co-twin samples) were extracted from saliva and buccal cheek swab samples and hybridized to HumanOmniExpressExome-8v1.2 genotyping arrays at the Institute of Psychiatry, Psychology and Neuroscience Genomics & Biomarker Core Facility. The raw image data from the array were normalized, pre-processed, and filtered in GenomeStudio according to Illumina Exome Chip SOP v1.4. (http://confluence.brc.iop.kcl.ac.uk:8090/display/PUB/Production+Version%3A+Illumina+Exome+Chip+SOP+v1.4). In addition, prior to genotype calling, 919 multi-mapping SNPs and 501 samples with callrate <0.95 were removed. The ZCALL program was used to augment the genotype calling for samples and SNPs that passed the initial quality control.

After initial quality control and genotype calling, the same quality control was performed on the samples genotyped on the Illumina and Affymetrix platforms separately using PLINK[18,19], R[20], BCFtools[21], and EIGENSOFT[22,23]. Specifically, samples were removed from subsequent analyses on the basis of call rate (< 0.98), suspected non-European ancestry, heterozygosity, and relatedness other than dizygotic twin status. SNPs were excluded if the minor allele frequency was < 0.5%, if > 2% of genotype data were missing, or if the HWE p-value was < $10^{-5}$. Non-autosomal markers and indels were removed. Association between SNP and the platform, batch, plate or well on which samples were genotyped was calculated; SNPs with an effect p-value < $10^{-4}$ were excluded. A total sample of 10,346 samples (including 3,320 dizygotic twin pairs), with 7,289 individuals and 559,772 SNPs genotyped on Illumina and 3,057 individuals and 635,269 SNPs genotyped on Affymetrix remained after quality control.

Genotypes from the two platforms were separately phased using EAGLE2[24], and imputed into the Haplotype Reference Consortium (release 1.1) using the Positional Burrows-Wheeler Transform method[25] through the Sanger Imputation Service[26]. Prior to merging, variants were excluded if INFO < 0.75 or were non-overlapping between platforms. Post-merge, SNPs with MAF ($p < 10^{-4)}$ and HWE ($p > 10^{-5}$) differences between platforms were removed. Using these criteria, 7,363,646 genotyped and well-imputed SNPs were retained.

Principal component analysis was performed on a subset of 39,353 common (MAF > 5%), perfectly imputed (INFO = 1) autosomal SNPs, after stringent pruning to remove markers in linkage disequilibrium ($r^2 > 0.1$) and excluding high linkage disequilibrium genomic regions so as to ensure that only genome-wide effects were detected.

**Supplementary note 4**: Estimation of genetic correlations

Details of the GWAS sumstat datasets used in genetic correlation analyses. Genetic correlations were conducted using linkage disequilibrium score regression (LDSR; http://ldsc.broadinstitute.org). We ran pair-wise genetic correlations with 13 psychiatric, cognitive and psychiatric and personality traits using GWAS summary statistics. A summary of each of the studies is provided below.

The meta-analysis GWAS on lifespan **intelligence** included 78,308 individuals from 13 different cohorts of European descent: eight cohorts of children <18 years (N =19,509), and five cohorts of adults 18 –78 years (N =58,799). The measure of intelligence for these cohorts was either ‘g’ or a primary measure of fluid intelligence [4]. The study on **childhood intelligence** includes a child-only subsample (6–18 years) of 17,989 individuals (N discovery=12,441) also included in the Sniekers et al. study, again using a combination of g and fluid intelligence measures [5]. **Years in education** is derived from the 2016 Social Science Genetic Association Consortium (SSGAC) meta-analysis GWAS that included adults (<30 years) of European descent (N discovery = 293,723; N replication = 405,072). The measure of educational attainment used in this study was number of years spent in education [6]. **Subjective well-being**, **depressive symptoms** and **neuroticism** were all reported in a single study and measured using a range of survey-based questions harmonised across cohorts. Subjective well-being was a meta-analysis GWAS of 59 cohorts totalling 298,420 individuals. Depressive symptoms (N = 161,460), and neuroticism (N = 170,910) GWAS were performed by combining publicly available summary statistics from published studies and new genome-wide analyses of additional data [7]. The **anorexia** summary statistics were taken from a meta-analysis of 5,551 cases with and 21,080 controls as part of the Genetic Consortium for Anorexia Nervosa (GCAN) and the Wellcome Trust Case Control Consortium 3 (WTCCC3) [8]. The major depressive disorder (**MDD)** GWAS required affected individuals to have a diagnosis of DSM-IV lifetime MDD established using structured diagnostic instruments from direct

interviews by trained interviewers or clinician-administered DSM-IV checklists. Controls were randomly selected from the population and screened for lifetime history of MDD. The discovery mega-analysis consisted of 18,759 independent and unrelated subjects (9,240 MDD cases and 9,519 controls) [9].

The **ADHD** GWAS, performed by the EArly Genetics and Lifecourse Epidemiology (EAGLE) consortium, genome-wide single nucleotide polymorphisms (SNPs) and ADHD symptom scores were available for 17,666 children (< 13 years) from nine population-based cohorts. SNP-based heritability was estimated in data from the three largest cohorts. Meta-analysis based on genome-wide association analyses with SNPs was followed by gene-based association tests, and the overlap in results with a meta-analysis in the Psychiatric Genomics Consortium (PGC) case-control ADHD study was investigated [10]. A meta-analysis of genome-wide association data was performed for the personality traits **Openness to Experience**, and **Conscientiousness** personality in ten discovery samples (17,375 adults) and five in-silico replication samples (3 294 adults). All participants were of European ancestry. Personality scores were based on the NEO Five-Factor Inventory [12]. The GWAS for those affected with **bipolar disorder** included 7,481 cases from 11 different groups with European ancestry and 9,250 control individuals [13].

## Supplementary Tables

**Table S2:** GWAS results for the SNPs in LD with rs9529641.

| rsID | pos | MAF | P | beta | se | r2 | nearestGene |
|---|---|---|---|---|---|---|---|
| rs9529643 | 35321101 | 0.2813 | 5.61E-08 | 0.115 | 0.021 | 0.990 | LINC00457 |
| rs9542089 | 35319024 | 0.2793 | 6.07E-08 | 0.114 | 0.021 | 1 | LINC00457 |
| rs9542077 | 35316237 | 0.2793 | 7.80E-08 | 0.114 | 0.021 | 1 | LINC00457 |
| rs1605645 | 35300769 | 0.2813 | 1.51E-06 | 0.101 | 0.021 | 0.921 | LINC00457 |
| rs1588227 | 35301279 | 0.2813 | 1.54E-06 | 0.101 | 0.021 | 0.921 | LINC00457 |
| rs1605644 | 35300861 | 0.2813 | 1.54E-06 | 0.101 | 0.021 | 0.921 | LINC00457 |
| rs9541997 | 35302796 | 0.2813 | 1.55E-06 | 0.101 | 0.021 | 0.921 | LINC00457 |
| rs1828145 | 35300416 | 0.2813 | 1.55E-06 | 0.101 | 0.021 | 0.921 | LINC00457 |
| rs3915162 | 35300017 | 0.2813 | 1.59E-06 | 0.101 | 0.021 | 0.921 | LINC00457 |
| rs3858857 | 35306267 | 0.2823 | 1.85E-06 | 0.100 | 0.021 | 0.917 | LINC00457 |
| rs1588228 | 35300287 | 0.2813 | 1.86E-06 | 0.100 | 0.021 | 0.921 | LINC00457 |
| rs2202780 | 35295773 | 0.2813 | 1.97E-06 | 0.100 | 0.021 | 0.921 | LINC00457 |
| rs9529598 | 35295159 | 0.2813 | 1.99E-06 | 0.100 | 0.021 | 0.921 | LINC00457 |
| rs7317557 | 35304127 | 0.2813 | 2.00E-06 | 0.099 | 0.021 | 0.921 | LINC00457 |
| rs9542020 | 35307592 | 0.2843 | 2.01E-06 | 0.099 | 0.021 | 0.909 | LINC00457 |
| rs1396308 | 35307838 | 0.2813 | 2.03E-06 | 0.100 | 0.021 | 0.903 | LINC00457 |
| rs7333541 | 35294988 | 0.2813 | 2.04E-06 | 0.100 | 0.021 | 0.921 | LINC00457 |
| rs9542019 | 35307444 | 0.2843 | 2.07E-06 | -0.099 | 0.021 | 0.909 | LINC00457 |
| rs9542025 | 35309058 | 0.2843 | 2.10E-06 | 0.099 | 0.021 | 0.909 | LINC00457 |
| rs12877643 | 35314360 | 0.2704 | 2.11E-06 | 0.102 | 0.022 | 0.870 | LINC00457 |
| rs7324868 | 35308074 | 0.2843 | 2.11E-06 | 0.099 | 0.021 | 0.909 | LINC00457 |
| rs1396309 | 35296712 | 0.2813 | 2.15E-06 | 0.099 | 0.021 | 0.921 | LINC00457 |
| rs73165804 | 35294254 | 0.2813 | 2.17E-06 | 0.099 | 0.021 | 0.921 | LINC00457 |
| rs9541976 | 35297304 | 0.2813 | 2.18E-06 | 0.099 | 0.021 | 0.921 | LINC00457 |
| rs9541975 | 35297268 | 0.2813 | 2.18E-06 | 0.099 | 0.021 | 0.921 | LINC00457 |
| rs1396307 | 35307848 | 0.2823 | 2.23E-06 | 0.099 | 0.021 | 0.899 | LINC00457 |
| rs2048198 | 35310197 | 0.2833 | 2.30E-06 | -0.099 | 0.021 | 0.913 | LINC00457 |
| rs1508487 | 35293969 | 0.2813 | 2.88E-06 | 0.098 | 0.021 | 0.921 | LINC00457 |

**Table S3**: LD-clumped genome-wide significant (bolded: $p \leq 5 \times 10^{-8}$) and suggestive ($p \leq 1 \times 10^{-5}$) SNP associations with science attainment.

| Chr | Allele (non-effect:effect) | rsid | Base position | INFO | MAF | Beta | SE | p-value | Gene |
|---|---|---|---|---|---|---|---|---|---|
| 13 | C:A | **rs9529641** | **35319175** | 0.991 | **0.250** | **0.1152** | **0.021** | **$4.86 \times 10^{-8}$** | ***LINC00457**** |
| 1 | A:G | rs6688495 | 103204647 | 0.982 | 0.152 | -0.117 | 0.026 | $4.93 \times 10^{-6}$ | COL11A1* |
| 1 | G:A | rs10797090 | 153624791 | 0.999 | 0.461 | -0.083 | 0.018 | $4.98 \times 10^{-6}$ | CHTOP* |
| 1 | G:A | rs11264236 | 153658480 | 0.922 | 0.291 | 0.099 | 0.021 | $2.46 \times 10^{-6}$ | NPR1** |
| 3 | C:T | rs78393327 | 174079287 | 0.991 | 0.019 | -0.325 | 0.066 | $1.01 \times 10^{-6}$ | NLGN1* |
| 3 | C:G | rs180928518 | 192643866 | 0.737 | 0.013 | 0.418 | 0.094 | $8.55 \times 10^{-6}$ | MB21D2* |
| 5 | C:T | rs27565 | 59837591 | 0.952 | 0.476 | 0.084 | 0.019 | $7.09 \times 10^{-6}$ | PART1** |
| 5 | C:T | rs13158665 | 60069057 | 0.997 | 0.457 | -0.085 | 0.018 | $3.72 \times 10^{-6}$ | ELOVL7** |
| 5 | A:G | rs1444240 | 60069934 | 0.998 | 0.439 | -0.089 | 0.019 | $1.59 \times 10^{-6}$ | ELOVL7** |
| 5 | A:G | rs10462335 | 88088204 | 0.999 | 0.479 | -0.089 | 0.018 | $7.99 \times 10^{-7}$ | MEF2C** |
| 5 | C:T | rs13171212 | 92674287 | 0.841 | 0.324 | -0.097 | 0.021 | $3.46 \times 10^{-6}$ | NR2F1-AS1* |
| 5 | C:T | rs4869194 | 92675000 | 0.965 | 0.201 | -0.104 | 0.023 | $6.93 \times 10^{-6}$ | NR2F1-AS1* |
| 5 | A:G | rs264864 | 169061462 | 0.987 | 0.312 | 0.097 | 0.020 | $7.21 \times 10^{-7}$ | DOCK2** |
| 8 | A:G | rs10100356 | 130626164 | 0.989 | 0.226 | -0.099 | 0.022 | $4.95 \times 10^{-6}$ | CCDC26** |
| 8 | A:G | rs4736679 | 134499912 | 0.987 | 0.057 | 0.177 | 0.039 | $5.31 \times 10^{-6}$ | ST3GAL1** |
| 8 | C:T | rs1554968 | 134507904 | 0.970 | 0.106 | 0.147 | 0.029 | $6.37 \times 10^{-7}$ | ST3GAL1** |
| 10 | C:T | rs10905791 | 5688085 | 0.961 | 0.394 | 0.091 | 0.019 | $1.58 \times 10^{-6}$ | ASB13** |
| 11 | A:G | rs11603691 | 57107617 | 0.995 | 0.115 | -0.133 | 0.028 | $3.02 \times 10^{-6}$ | P2RX3** |
| 14 | C:T | rs2415955 | 46803675 | 0.996 | 0.435 | -0.081 | 0.018 | $7.35 \times 10^{-6}$ | LINC00871** |
| 14 | A:G | rs7141746 | 94993936 | 0.805 | 0.420 | -0.102 | 0.019 | $1.22 \times 10^{-7}$ | SERPINA12* |
| 16 | A:G | rs237149 | 26649640 | 0.995 | 0.401 | -0.082 | 0.018 | $9.94 \times 10^{-6}$ | HS3ST4* |
| 17 | C:T | rs2529909 | 13928634 | 0.711 | 0.164 | 0.127 | 0.028 | $7.66 \times 10^{-6}$ | COX10-AS1** |
| 17 | A:G | rs35751000 | 34913528 | 0.998 | 0.419 | 0.082 | 0.018 | $9.25 \times 10^{-6}$ | GGNBP2** |
| 19 | A:G | rs35681564 | 43959388 | 0.960 | 0.188 | -0.104 | 0.024 | $9.54 \times 10^{-6}$ | LYPD3* |
| 19 | C:T | rs62133140 | 54773032 | 0.912 | 0.498 | -0.087 | 0.019 | $4.80 \times 10^{-6}$ | LILRB2* |
| 20 | C:T | rs28579792 | 59937459 | 0.913 | 0.395 | -0.091 | 0.020 | $3.37 \times 10^{-6}$ | CDH4** |
| 21 | C:T | rs35739539 | 18803733 | 0.879 | 0.110 | 0.145 | 0.031 | $3.01 \times 10^{-6}$ | RNU6-113P* |

*Nearest gene, ** In gene according to the NCBI website database

**Table S4**: LD-clumped suggestive ($p \leq 1 \times 10^{-5}$) SNP associations with maths attainment.

| Chr | Allele (non-effect:effect) | rsid | Base position | Info | MAF | Beta | SE | p-value | Gene |
|---|---|---|---|---|---|---|---|---|---|
| 1 | A:G | rs1572040 | 3337543 | 0.914 | 0.119 | -0.157 | 0.029 | $7.77 \times 10^{-8}$ | PRDM16** |
| 1 | C:T | rs148442247 | 67984971 | 0.905 | 0.018 | 0.325 | 0.072 | $6.41 \times 10^{-6}$ | SERBP1* |
| 2 | C:T | rs72815377 | 63599530 | 0.998 | 0.208 | 0.104 | 0.022 | $3.78 \times 10^{-6}$ | WDPCP** |
| 2 | A:T | rs138004766 | 107911721 | 0.963 | 0.050 | 0.190 | 0.043 | $9.56 \times 10^{-6}$ | AC006227.1** |
| 3 | G:T | rs145032667 | 39361044 | 0.973 | 0.018 | 0.310 | 0.070 | $9.03 \times 10^{-6}$ | CX3CR1* |
| 4 | A:T | rs11099631 | 84901818 | 0.983 | 0.019 | -0.322 | 0.072 | $9.37 \times 10^{-6}$ | RP11-8L2.1** |
| 4 | A:C | rs72699806 | 169552640 | 0.885 | 0.222 | -0.098 | 0.022 | $6.67 \times 10^{-6}$ | PALLD** |
| 5 | A:T | rs55962375 | 10084607 | 0.981 | 0.019 | -0.323 | 0.067 | $1.54 \times 10^{-6}$ | FAM173B* |
| 5 | A:G | rs138210978 | 37748954 | 0.994 | 0.311 | 0.094 | 0.020 | $5.54 \times 10^{-6}$ | WDR70** |
| 5 | A:G | rs2961857 | 165947835 | 0.983 | 0.270 | 0.093 | 0.021 | $7.06 \times 10^{-6}$ | CTB-7E3.1* |
| 5 | A:G | rs264864 | 169061462 | 0.987 | 0.220 | -0.100 | 0.022 | $1.85 \times 10^{-6}$ | DOCK2* |
| 6 | C:G | rs227479 | 165445123 | 0.995 | 0.382 | 0.088 | 0.019 | $2.79 \times 10^{-6}$ | C5orf118* |
| 7 | A:G | rs78700728 | 81501789 | 0.986 | 0.329 | 0.090 | 0.020 | $7.51 \times 10^{-6}$ | CACNA2D1* |
| 7 | C:T | rs75215979 | 111308302 | 0.772 | 0.038 | 0.216 | 0.048 | $9.31 \times 10^{-6}$ | DOCK4* |
| 7 | A:C | rs4732313 | 138091839 | 0.994 | 0.033 | 0.256 | 0.058 | $3.78 \times 10^{-6}$ | TRIM24* |
| 8 | C:T | rs60626139 | 6503455 | 0.962 | 0.331 | -0.093 | 0.020 | $5.17 \times 10^{-6}$ | MCPH1* |
| 8 | C:T | rs66477371 | 18313717 | 0.968 | 0.067 | -0.170 | 0.037 | $5.85 \times 10^{-6}$ | NAT2* |
| 8 | C:G | rs55780284 | 39989212 | 0.984 | 0.033 | 0.236 | 0.052 | $1.73 \times 10^{-6}$ | C8orf4* |
| 8 | A:G | rs4961372 | 142358865 | 0.647 | 0.183 | -0.128 | 0.029 | $8.71 \times 10^{-6}$ | GPR20* |
| 9 | C:T | rs10780702 | 72537085 | 0.999 | 0.222 | -0.170 | 0.029 | $8.37 \times 10^{-7}$ | C9orf135* |
| 9 | C:T | rs10780705 | 72539240 | 0.997 | 0.287 | 0.100 | 0.020 | $6.21 \times 10^{-6}$ | C9orf135* |
| 10 | C:G | rs10795666 | 8686904 | 0.991 | 0.099 | -0.189 | 0.041 | $9.34 \times 10^{-6}$ | CHCHD3P1* |
| 10 | A:G | rs10822834 | 68224205 | 0.943 | 0.029 | -0.251 | 0.056 | $9.08 \times 10^{-6}$ | CTNNA3** |
| 11 | C:G | rs7129491 | 57095082 | 1.000 | 0.114 | -0.138 | 0.028 | $1.31 \times 10^{-6}$ | SSRP1** |
| 11 | C:G | rs1504712 | 99240470 | 0.994 | 0.497 | -0.084 | 0.018 | $4.50 \times 10^{-6}$ | CNTN5** |
| 12 | A:G | rs7139245 | 2955636 | 0.684 | 0.170 | -0.157 | 0.028 | $2.68 \times 10^{-8}$ | ITFG2** |
| 12 | A:G | rs74398913 | 4027686 | 0.837 | 0.266 | 0.098 | 0.021 | $2.69 \times 10^{-6}$ | PARP11* |
| 12 | A:G | rs11545332 | 31256995 | 0.977 | 0.031 | 0.270 | 0.057 | $2.55 \times 10^{-6}$ | DDX11** |
| 12 | A:G | rs10744264 | 126967956 | 0.875 | 0.100 | -0.145 | 0.032 | $6.44 \times 10^{-6}$ | RP5-944M2.3* |
| 13 | C:T | rs9541641 | 69524675 | 0.998 | 0.451 | -0.087 | 0.019 | $3.30 \times 10^{-6}$ | ZDHHC20P4* |
| 14 | A:G | rs7141746 | 94993936 | 0.804 | 0.420 | -0.097 | 0.019 | $5.29 \times 10^{-7}$ | SERPINA12* |
| 15 | A:C | rs7182195 | 53027917 | 0.975 | 0.019 | -0.344 | 0.068 | $4.87 \times 10^{-7}$ | ONECUT1* |
| 16 | A:G | rs72797219 | 83715276 | 0.992 | 0.178 | 0.114 | 0.024 | $2.10 \times 10^{-6}$ | CDH13** |
| 16 | C:T | rs118158969 | 84404400 | 0.990 | 0.106 | -0.138 | 0.030 | $4.04 \times 10^{-6}$ | ATP2C2** |
| 18 | C:T | rs12967053 | 47635907 | 0.994 | 0.160 | -0.156 | 0.033 | $5.23 \times 10^{-6}$ | MYO5B** |
| 20 | A:T | rs146283583 | 2736559 | 0.828 | 0.019 | 0.326 | 0.071 | $5.06 \times 10^{-6}$ | EBF4** |
| 21 | C:T | rs66795148 | 24356507 | 0.989 | 0.366 | 0.086 | 0.019 | $6.96 \times 10^{-6}$ | ZNF299P* |
| 22 | C:T | rs62219796 | 23631823 | 0.995 | 0.277 | 0.090 | 0.020 | $9.80 \times 10^{-6}$ | BCR** |

*Nearest gene, ** In gene according to the NCBI website database

**Table S5**: LD-clumped suggestive ($p \leq 1 \times 10^{-5}$) SNP associations with attainment in English.

| Chr | Allele (non-effect:effect) | rsid | Base position | Info | MAF | Beta | SE | p-value | Gene |
|---|---|---|---|---|---|---|---|---|---|
| 1 | A:G | rs77176926 | 88142569 | 0.813 | 0.021 | -0.324 | 0.070 | $3.82 \times 10^{-6}$ | LMO4* |
| 2 | A:G | rs145957568 | 63454007 | 0.888 | 0.016 | -0.371 | 0.076 | $1.17 \times 10^{-6}$ | WDPCP** |
| 3 | C:T | rs6767406 | 23862463 | 0.998 | 0.443 | -0.088 | 0.019 | $7.00 \times 10^{-6}$ | UBE2E1** |
| 3 | A:T | rs6779537 | 23901028 | 0.998 | 0.258 | -0.096 | 0.021 | $1.85 \times 10^{-6}$ | UBE2E1** |
| 3 | A:G | rs35576002 | 150212583 | 0.986 | 0.486 | -0.083 | 0.018 | $4.67 \times 10^{-6}$ | SERP1* |
| 4 | G:T | rs75596547 | 140729403 | 0.890 | 0.030 | -0.259 | 0.057 | $4.65 \times 10^{-6}$ | MAML3** |
| 5 | C:T | rs13188649 | 103467631 | 0.922 | 0.016 | 0.356 | 0.075 | $2.11 \times 10^{-6}$ | NUDT12* |
| 7 | C:T | rs118066386 | 158839899 | 0.819 | 0.024 | 0.289 | 0.065 | $8.45 \times 10^{-6}$ | VIPR2** |
| 8 | C:T | rs35112066 | 34289939 | 0.991 | 0.097 | 0.149 | 0.031 | $1.79 \times 10^{-6}$ | Rp11-258J101* |
| 8 | C:G | rs147918758 | 41167562 | 0.986 | 0.390 | -0.084 | 0.019 | $7.59 \times 10^{-6}$ | SFRP1* |
| 11 | C:T | rs36234212 | 17499694 | 0.914 | 0.045 | -0.206 | 0.046 | $7.27 \times 10^{-6}$ | ABCC8* |
| 12 | G:T | rs7305366 | 43577713 | 0.998 | 0.459 | -0.081 | 0.018 | $9.46 \times 10^{-6}$ | ADAMTS20* |
| 14 | A:T | rs9707389 | 30535726 | 0.997 | 0.018 | 0.332 | 0.069 | $1.42 \times 10^{-6}$ | PRKD1** |
| 15 | C:G | rs12441039 | 36326018 | 0.993 | 0.131 | -0.127 | 0.027 | $2.78 \times 10^{-6}$ | MIR450* |
| 19 | C:T | rs117322896 | 11979669 | 0.975 | 0.013 | 0.384 | 0.082 | $2.70 \times 10^{-6}$ | ZNF439** |
| 20 | C:T | rs115416341 | 46471358 | 0.988 | 0.025 | 0.276 | 0.059 | $2.48 \times 10^{-6}$ | SULF2* |

*Nearest gene, ** In gene according to the NCBI website database

**Table S6**: MAGMA gene-set analysis.

| Phenotype | Gene Set | N genes | Beta | Beta STD | SE | P | P bonferroni |
|---|---|---|---|---|---|---|---|
| Science | GO bp: go negative regulation of locomotion | 244 | 0.255 | 0.030 | 0.052 | 4.82x10-7 | 0.005 |
| English | GO bp: go negative regulation of calcium ion dependent exocytosis | 9 | 1.457 | 0.033 | 0.284 | 1.42x10-7 | 0.002 |
| English | GO bp: go negative regulation of_exocytosis | 24 | 0.788 | 0.029 | 0.178 | 4.94x10-6 | 0.053 |

Competitive MAGMA gene-set analyses using 10,673 gene sets. The table displays significant gene sets with $P_{bon} \leq 0.05$. P-values are Bonferroni adjusted in accordance with the number of gene-sets tested (0.05/10,673). GO bp are a set of genes from the Gene Ontology Resource under the category biological processes (other categories included cellular components and molecular functions).

**Table S7**: Genome-wide heritability estimate comparisons between Linkage Disequilibrium Score (LDSC) regression constrained, unconstrained and genome-based restricted maximum likelihood (GREML) estimates. These analyses were carried out for each subject attainment measure and repeated regressing out the other two attainment measures (AA-English, AA-Maths, AA-Science) or IQ (IQ-English, IQ-Maths, IQ-Science).

| $h^2_{SNP}$ | LDSC (unconstrained) | LDSC (constrained) | GREML |
|---|---|---|---|
| English | 0.285 (0.086)* | 0.360 (0.058) | 0.412 (0.059) |
| Maths | 0.337 (0.087)* | 0.473 (0.058) | 0.472 (0.058) |
| Science | 0.313 (0.090)* | 0.535 (0.058) | 0.532 (0.057) |
| English AAreg | 0.025 (0.091) | 0.057 (0.057) | 0.077 (0.058) |
| Maths AAreg | 0.072 (0.086) | 0.117 (0.058) | 0.100 (0.058) |
| Science AAreg | 0.062 (0.083) | 0.126 (0.054) | 0.190 (0.060) |
| English IQreg | 0.028 (0.148) | 0.065 (0.101) | 0.132 (0.107) |
| Maths IQreg | 0.042 (0.150)* | 0.244 (0.107) | 0.207 (0.109) |
| Science IQreg | 0.117 (0.135) | 0.145 (0.093) | 0.123 (0.105) |

*LD intercept is significantly larger than 1. Standard errors are in the parentheses.

**Table S8:** LD score genetic correlations between English, maths and science attainment, and the 13 selected educational, cognitive and psychological traits. Bolded text shows significant associations at a Bonferroni correction threshold of p ≤ 0.001 (0.05/39).

| | Trait2 (PMID) | pop ancestry | Trait h2 (se) | Trait h2 intercept (se) | Trait 1 | rg (se) | z | p | Genetic covariance intercept (se) |
|---|---|---|---|---|---|---|---|---|---|
| Cognitive | Intelligence (28530673) | Euro | 0.188 (0.011) | 1.005 (0.010) | **Science** | **1.24 (0.17)** | **7.19** | **$6.29 \times 10^{-13}$** | **0.028 (0.006)** |
| | | | | | **Maths** | **1.20 (0.16)** | **7.60** | **$3.02 \times 10^{-14}$** | **0.035 (0.006)** |
| | | | | | **English** | **1.26 (0.19)** | **6.72** | **$1.84 \times 10^{-11}$** | **0.020 (0.006)** |
| | Childhood IQ (23358156) | Euro | 0.305 (0.050) | 0.993 (0.011) | **Science** | **1.20 (0.21)** | **5.85** | **$4.92 \times 10^{-9}$** | **0.266 (0.008)** |
| | | | | | **Maths** | **1.02 (0.16)** | **6.38** | **$1.73 \times 10^{-10}$** | **0.270 (0.007)** |
| | | | | | **English** | **1.20 (0.24)** | **4.99** | **$6.09 \times 10^{-7}$** | **0.222 (0.008)** |
| Education | Years of schooling 2016 (27225129) | Euro | 0.125 (0.005) | 0.938 (0.013) | **Science** | **0.95 (0.13)** | **7.18** | **$6.73 \times 10^{-13}$** | **-0.005 (0.006)** |
| | | | | | **Maths** | **0.89 (0.12)** | **7.22** | **$5.35 \times 10^{-13}$** | **0.005 (0.007)** |
| | | | | | **English** | **0.99 (0.15)** | **6.63** | **$3.45 \times 10^{-11}$** | **-0.003 (0.006)** |
| Personality | Neo-openness to experience (21173776) | Euro | 0.105 (0.031) | 0.993 (0.008) | Science | 0.41 (0.21) | 1.95 | 0.0512 | 0.001 (0.006) |
| | | | | | Maths | 0.30 (0.2) | 1.50 | 0.1324 | -0.002 (0.006) |
| | | | | | English | 0.52 (0.23) | 2.23 | 0.0258 | -0.003 (0.006) |
| | Neo-conscientiousness (21173776) | Euro | 0.070 (0.033) | 1.001 (0.009) | Science | -0.25 (0.28) | -0.87 | 0.3859 | 0.001 (0.006) |
| | | | | | Maths | -0.02 (0.25) | -0.10 | 0.9240 | -0.005 (0.006) |
| | | | | | English | -0.14 (0.27) | -0.52 | 0.6012 | -0.006 (0.006) |
| | Neuroticism (27089181) | Euro | 0.089 (0.007) | 0.987 (0.014) | Science | -0.19 (0.09) | -2.10 | 0.0357 | 0(0.007) |
| | | | | | Maths | -0.17 (0.08) | -2.02 | 0.0432 | -0.001 (0.007) |
| | | | | | English | -0.1 (0.09) | -1.13 | 0.2597 | -0.003 (0.007) |
| | Subjective well-being (27089181) | Euro | 0.024 (0.002) | 1.006 (0.009) | Science | 0.08 (0.13) | 0.65 | 0.5134 | -0.001 (0.006) |
| | | | | | Maths | 0.10 (0.11) | 0.94 | 0.3447 | 0.003 (0.006) |
| | | | | | English | -0.12 (0.12) | -1.02 | 0.3097 | 0.009 (0.006) |
| Psychiatric | Autism spectrum disorder (0) | Euro | 0.454 (0.054) | 0.965 (0.008) | Science | 0.25 (0.12) | 1.97 | 0.0484 | 0.004 (0.005) |
| | | | | | **Maths** | **0.40 (0.12)** | **3.32** | **0.0009** | **0.001 (0.005)** |
| | | | | | English | 0.39 (0.14) | 2.90 | 0.0038 | -0.001 (0.006) |
| | Depressive symptoms (27089181) | Euro | 0.046 (0.004) | 1.002 (0.009) | **Science** | **-0.39 (0.12)** | **-3.27** | **0.0011** | **-0.001 (0.006)** |
| | | | | | **Maths** | **-0.37 (0.11)** | **-3.36** | **0.0008** | **-0.002 (0.006)** |
| | | | | | English | -0.27 (0.12) | -2.31 | 0.0212 | 0.001 (0.006) |
| | Bipolar disorder (21926972) | Euro | 0.445 (0.041) | 1.018 (0.009) | Science | 0.20 (0.13) | 1.48 | 0.1382 | -0.008 (0.006) |
| | | | | | Maths | 0.21 (0.11) | 1.83 | 0.0676 | -0.009 (0.006) |
| | | | | | English | 0.000 (0.14) | 1.42 | 0.1559 | -0.006 (0.006) |
| | Major depressive disorder (22472876) | Euro | 0.175 (0.031) | 1.004 (0.008) | Science | -0.13 (0.15) | -0.90 | 0.3662 | -0.008 (0.006) |
| | | | | | Maths | -0.17 (0.15) | -1.13 | 0.2565 | -0.008 (0.006) |

| | | | | | | | | |
|---|---|---|---|---|---|---|---|---|
| | | | | English | 0.04 (0.16) | 0.23 | 0.8212 | -0.003 (0.006) |
| ADHD (27663945) | Euro | 0.080 (0.031) | 0.992 (0.009) | Science | -0.77 (0.29) | -2.67 | 0.0076 | -0.103 (0.006) |
| | | | | Maths | -0.62 (0.26) | -2.41 | 0.0160 | -0.212 (0.006) |
| | | | | English | -0.72 (0.28) | -2.60 | 0.0094 | -0.118 (0.006) |
| Anorexia Nervosa (24514567) | Euro | 0.590 (0.032) | 0.868 (0.009) | Science | 0.04 (0.09) | 0.46 | 0.6432 | -0.002 (0.006) |
| | | | | Maths | 0.08 (0.09) | 0.91 | 0.3607 | -0.009 (0.006) |
| | | | | English | 0.28 (0.10) | 2.81 | 0.0050 | -0.012 (0.006) |

# Supplementary Figures

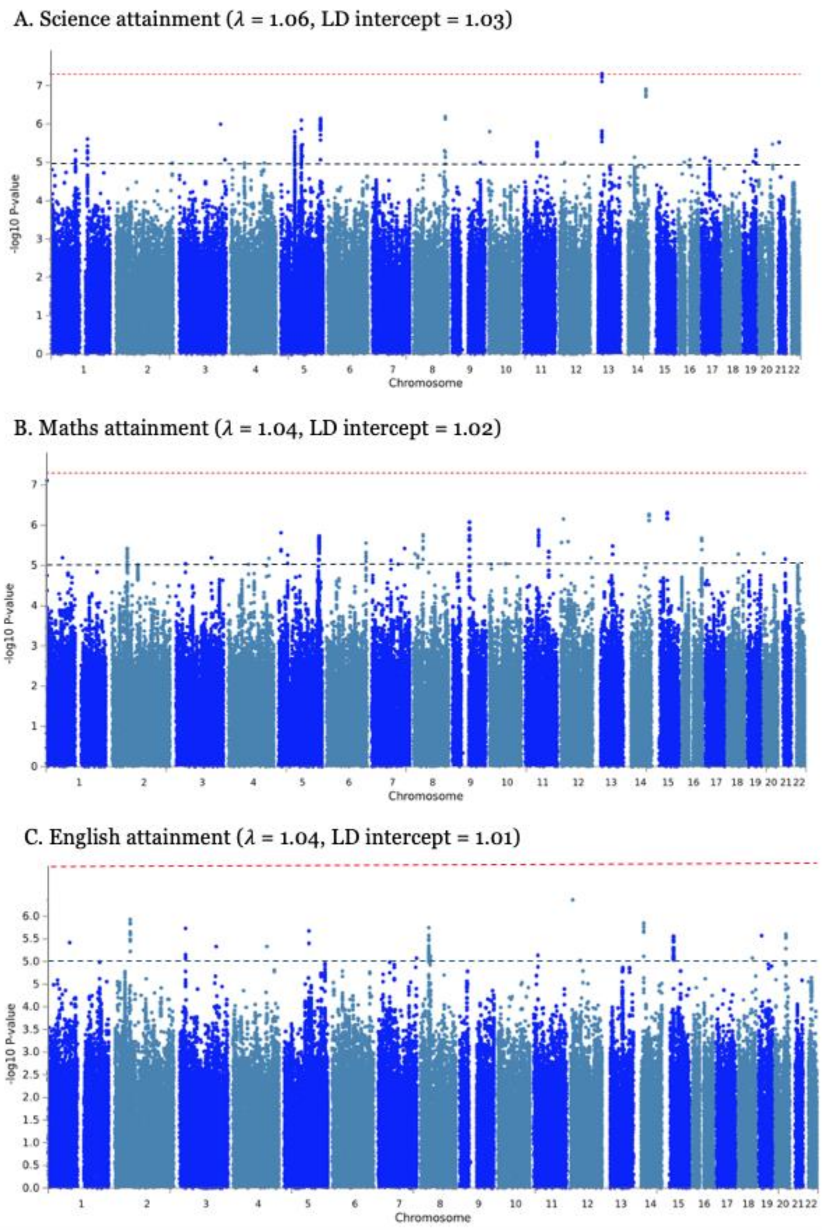


**Figure S1:** Manhattan plots of genome-wide association analyses for attainment in (A) science, (B) maths and (C) English. Dashed red line represents the genome-wide significant line ($p < 5 \times 10^{-8}$) and the blue dashed line is the suggestive line ($p < 1 \times 10^{-5}$).

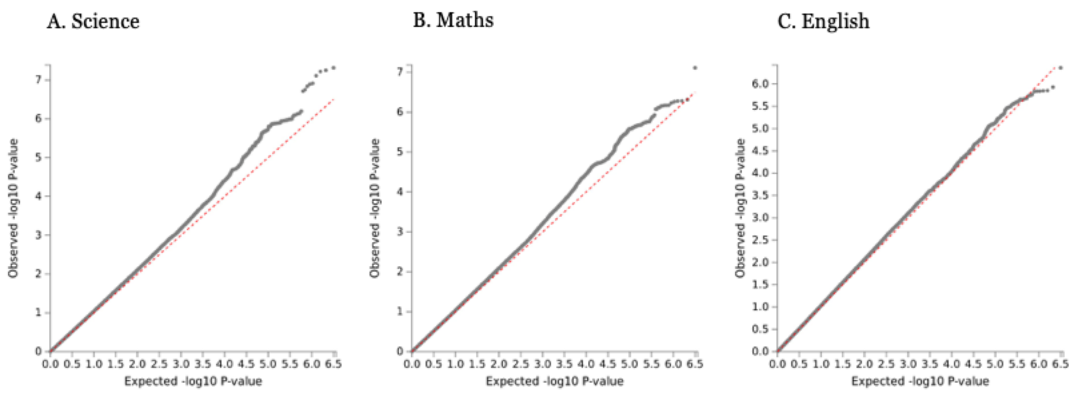


**Figure S2:** Quantile-quantile plots of genome-wide association analyses for attainment in (A) science, (B) maths and (C) English. The red dotted line represents the expected distribution of p-values under the null.

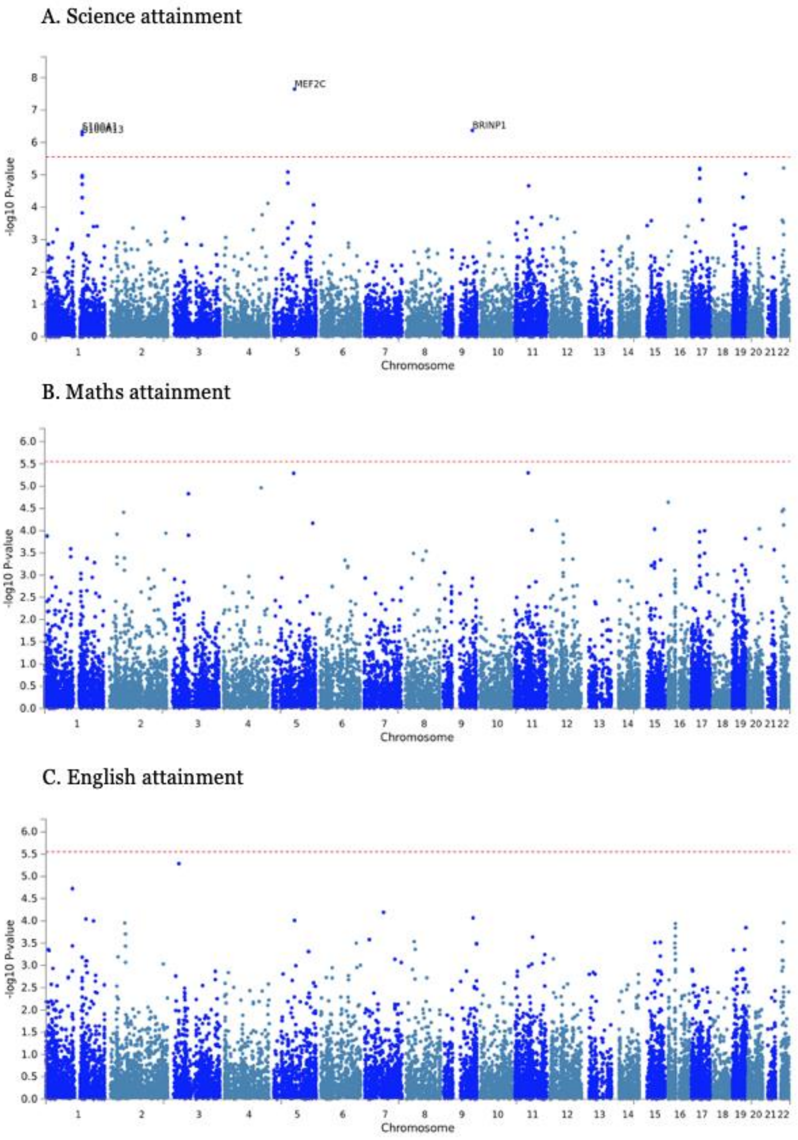


**Figure S3:** Manhattan plots of the gene-based associations analyses of (A) science, (B) maths and (C) English. Dashed red line represents Bonferroni-corrected genome-wide significance threshold ($p = 0.05/17875 = 2.3 \times 10^{-6}$).

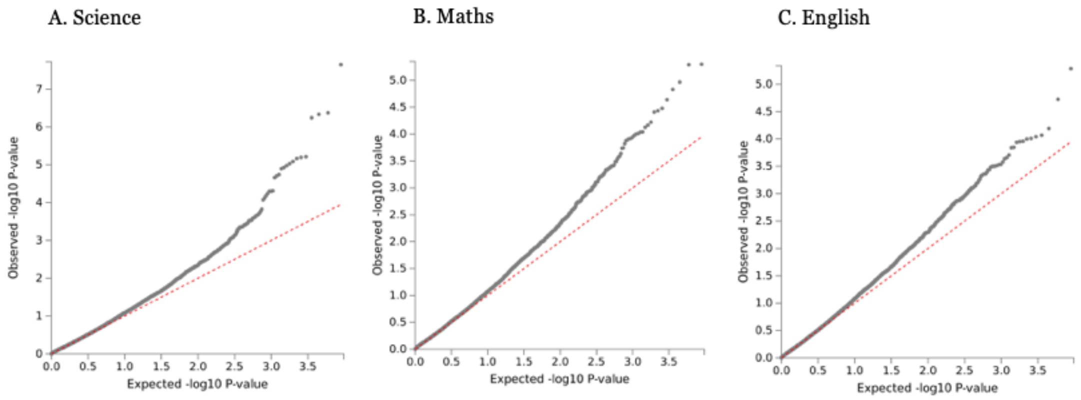


**Figure S4:** Quantile-quantile plots of the gene-based associations analyses for attainment in (A) science, (B) maths and (C) English. The red dotted line represents the expected distribution of p-values under the null.

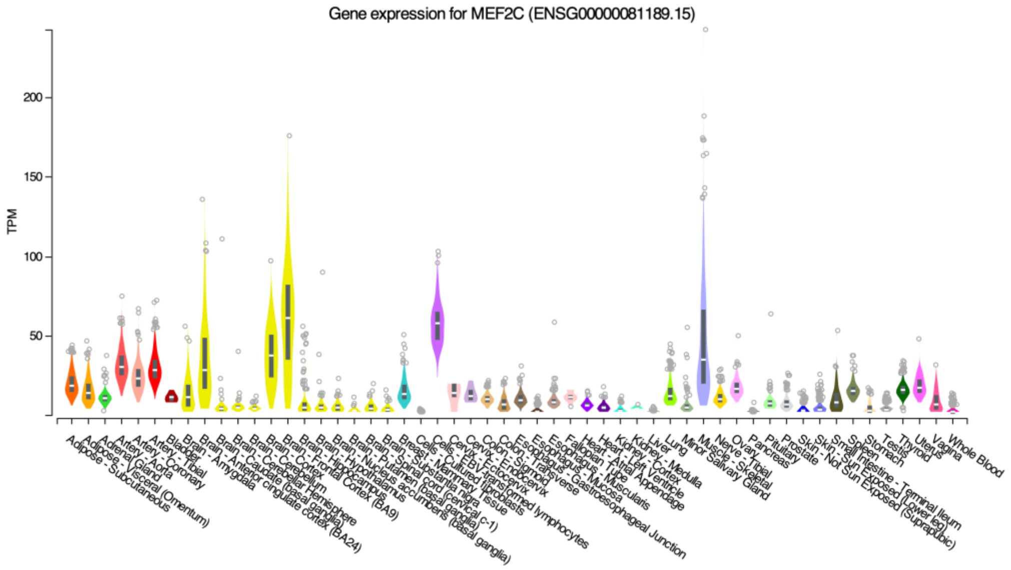


**Figure S5**: MEF2C gene expression data from GTEx Portal, sorted by tissue. TPM; Transcripts per Million.

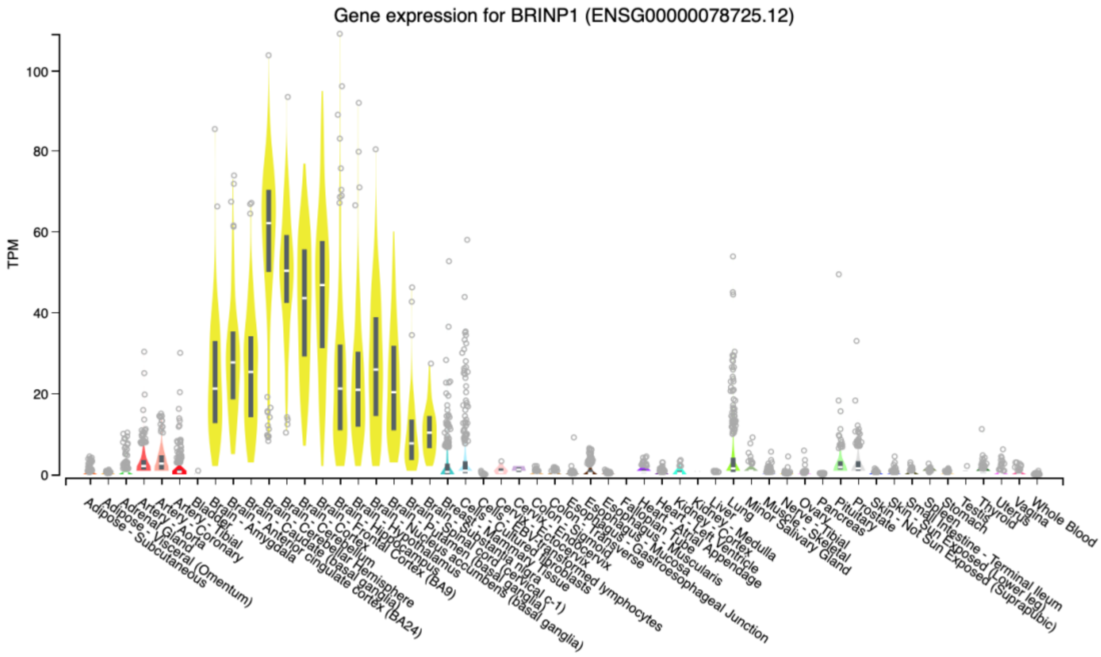


**Figure S6**: BRINP1 gene expression data from the GTEx Portal

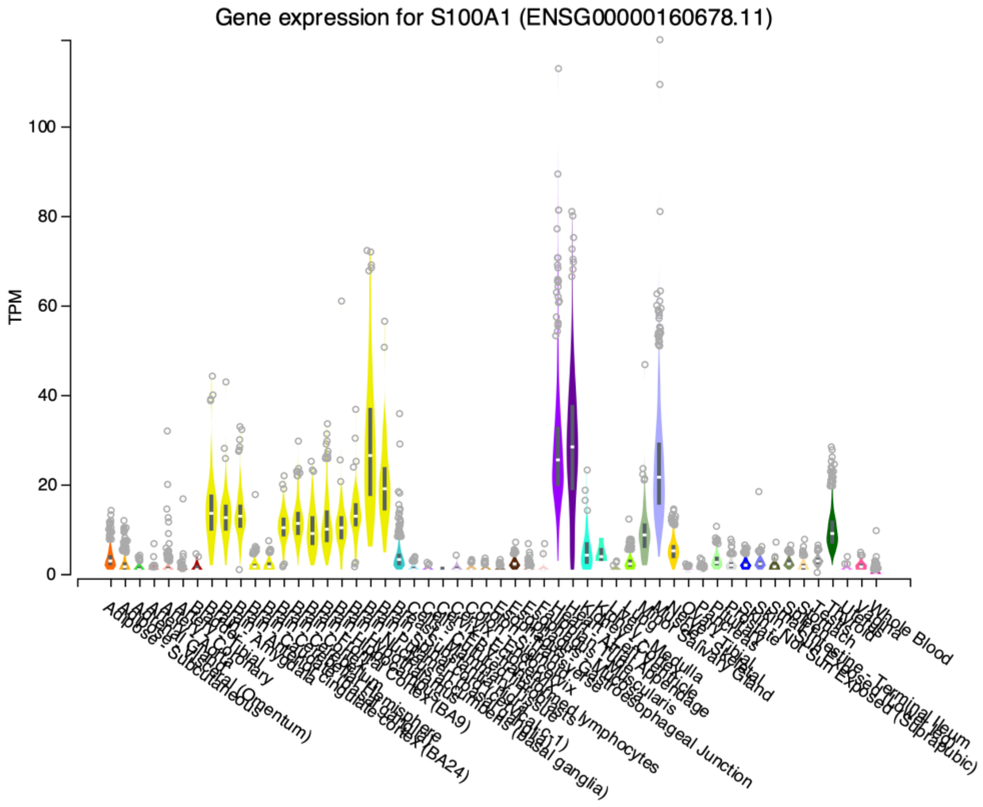


**Figure S7:** S100A1 gene expression data from the GTEx Portal

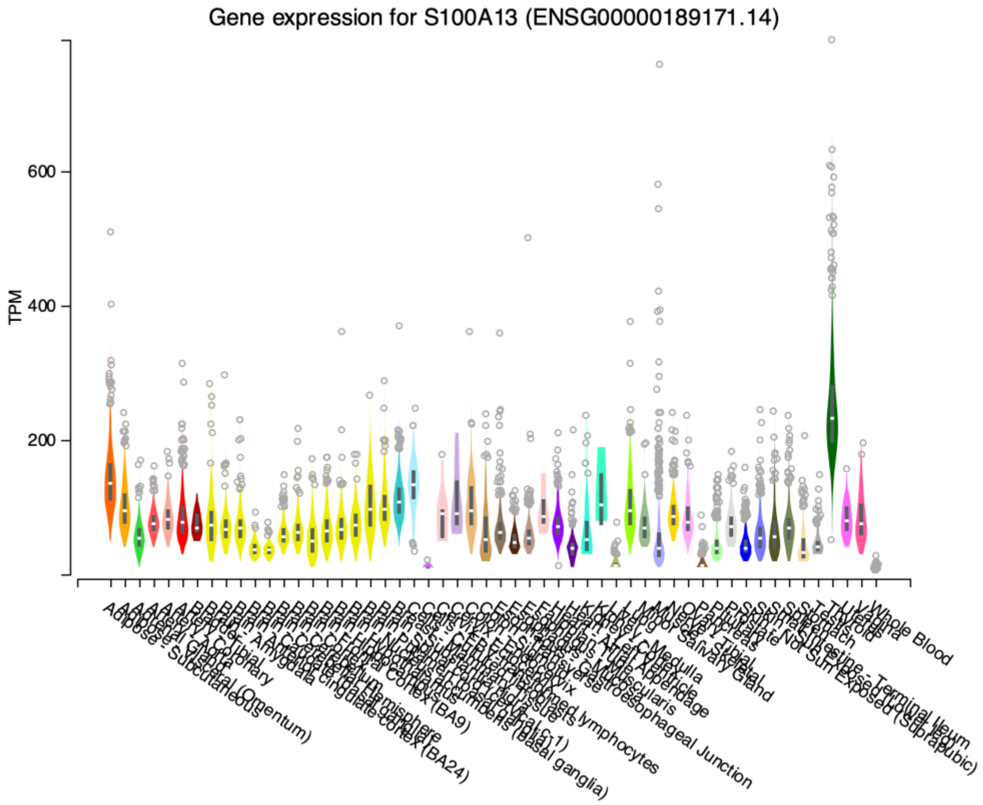


**Figure S8:** S100A13 gene expression data from the GTEx Portal

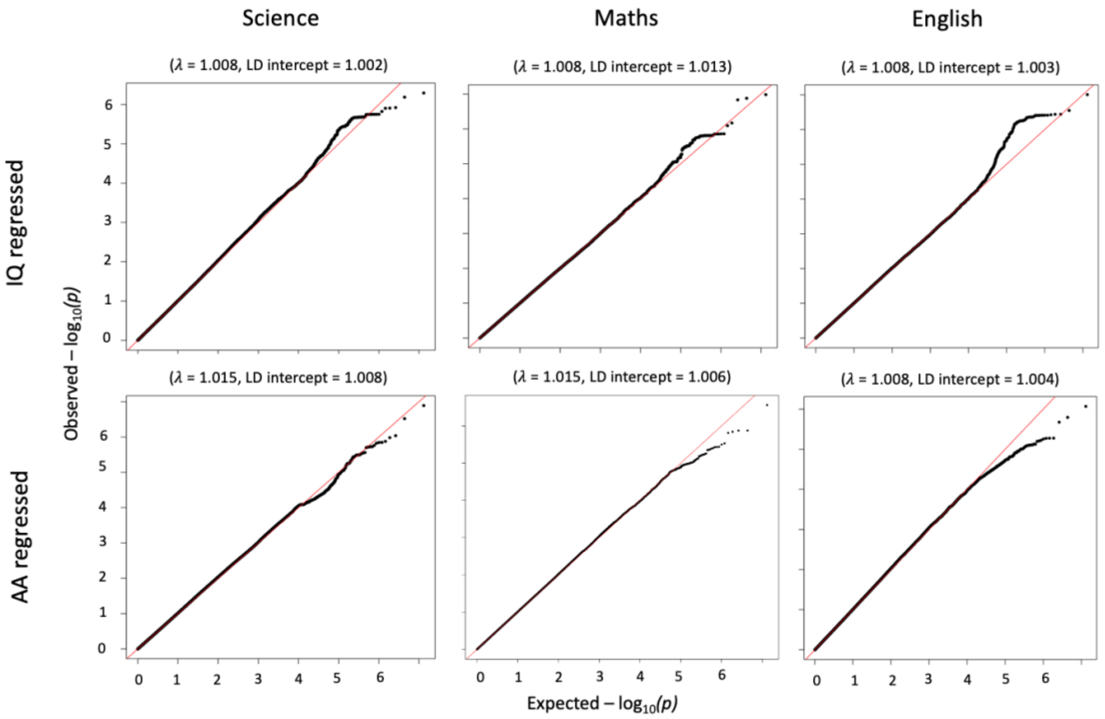


**Figure S9:** Quantile-quantile plots of the genome-wide associations analyses for attainment in science, maths and English regressing out IQ (top row) or attainment in the other two subjects (bottom row). The red dotted line represents the expected distribution of p-values under the null.

## Supplementary References


1. Boyd, A. *et al.* Cohort Profile: the 'children of the 90s'--the index offspring of the Avon Longitudinal Study of Parents and Children. *Int. J. Epidemiol.* **42**, 111–127 (2013).
2. Price, A. L. *et al.* Long-Range LD Can Confound Genome Scans in Admixed Populations. *Am. J. Hum. Genet.* **83**, 132–135 (2008).
3. Purcell, S. *et al.* PLINK: a tool set for whole-genome association and population-based linkage analyses. *Am. J. Hum. Genet.* **81**, 559–575 (2007).
4. Sniekers, S. *et al.* Genome-wide association meta-analysis of 78,308 individuals identifies new loci and genes influencing human intelligence. *Nat. Genet.* **49**, 1107–1112 (2017).
5. Benyamin, B. *et al.* Childhood intelligence is heritable, highly polygenic and associated with FNBP1L. *Mol. Psychiatry* **19**, 253–258 (2014).
6. Okbay, A. *et al.* Genome-wide association study identifies 74 loci associated with educational attainment. *Nature* **533**, 539–542 (2016).
7. Okbay, A. *et al.* Genetic variants associated with subjective well-being, depressive symptoms, and neuroticism identified through genome-wide analyses. *Nat. Genet.* **48**, 624–633 (2016).
8. The Wellcome Trust Case Control Consortium 322 *et al.* A genome-wide association study of anorexia nervosa. *Mol. Psychiatry* **19**, 1085–1094 (2014).
9. Major Depressive Disorder Working Group of the Psychiatric GWAS Consortium. A mega-analysis of genome-wide association studies for major depressive disorder. *Mol. Psychiatry* **18**, 497–511 (2013).

10. Middeldorp, C. M. *et al.* A Genome-Wide Association Meta-Analysis of Attention-Deficit/Hyperactivity Disorder Symptoms in Population-Based Paediatric Cohorts. 22 (2017).

11. Ripke, S. *et al.* Biological insights from 108 schizophrenia-associated genetic loci. *Nature* **511**, 421–427 (2014).

12. de Moor, M. H. M. *et al.* Meta-analysis of genome-wide association studies for personality. *Mol. Psychiatry* **17**, 337–349 (2012).

13. Psychiatric GWAS Consortium Bipolar Disorder Working Group. Large-scale genome-wide association analysis of bipolar disorder identifies a new susceptibility locus near ODZ4. *Nat. Genet.* **43**, 977–983 (2011).

14. Selzam, S. *et al.* Genome-Wide Polygenic Scores Predict Reading Performance Throughout the School Years. *Sci. Stud. Read.* **21**, 334–349 (2017).

15. Marchini, J., Howie, B., Myers, S., McVean, G. & Donnelly, P. A new multipoint method for genome-wide association studies by imputation of genotypes. *Nat. Genet.* **39**, 906–913 (2007).

16. Willer, C. J., Li, Y. & Abecasis, G. R. METAL: fast and efficient meta-analysis of genomewide association scans. *Bioinformatics* **26**, 2190–2191 (2010).

17. Leeuw, C. A. de, Mooij, J. M., Heskes, T. & Posthuma, D. MAGMA: Generalized Gene-Set Analysis of GWAS Data. *PLOS Comput. Biol.* **11**, e1004219 (2015).

18. Chang, C. C. *et al.* Second-generation PLINK: rising to the challenge of larger and richer datasets. *GigaScience* **4**, 7 (2015).

19. Purcell, S. *et al.* PLINK: A Tool Set for Whole-Genome Association and Population-Based Linkage Analyses. *Am. J. Hum. Genet.* **81**, 559–575 (2007).

20. R Core Team. R: A Language and Environment for Statistical Computing. *R Foundation for Statistical Computing* (2017).

21. Li, H. A statistical framework for SNP calling, mutation discovery, association mapping and population genetical parameter estimation from sequencing data. *Bioinformatics* **27**, 2987–2993 (2011).
22. Patterson, N., Price, A. L. & Reich, D. Population structure and eigenanalysis. *PLoS Genet.* **2**, e190 (2006).
23. Price, A. L. *et al.* Principal components analysis corrects for stratification in genome-wide association studies. *Nat. Genet.* **38**, 904 (2006).
24. Loh, P.-R. *et al.* Reference-based phasing using the Haplotype Reference Consortium panel. *Nat. Genet.* **48**, 1443–1448 (2016).
25. Durbin, R. Efficient haplotype matching and storage using the positional Burrows–Wheeler transform (PBWT). *Bioinformatics* **30**, 1266–1272 (2014).
26. McCarthy, S. *et al.* A reference panel of 64,976 haplotypes for genotype imputation. *bioRxiv* 035170 (2015).
